# Design and Implementation of a One Health Framework for Evaluating the Transmission Potential of Pathogens of Public Health Interest for Surveillance Studies

**DOI:** 10.1093/cid/ciaf487

**Published:** 2025-11-20

**Authors:** Claire A Quiner, Lauren P Courtney, Jean Kim, Vivian Kwaghe, Jay Osi Samuels, Cyril Erameh, Adamu Zigwai Ephraim, Emmanuel A Oga

**Affiliations:** Solutions, RTI International, Durham, North Carolina, USA; Solutions, RTI International, Durham, North Carolina, USA; Solutions, RTI International, Durham, North Carolina, USA; Internal Medicine, University of Abuja Teaching Hospital, Abuja, Nigeria; APIN Public Health Initiative, Abuja, Nigeria; Irrua Specialist Teaching Hospital, Edo, Nigeria; Solutions, RTI International, Durham, North Carolina, USA; Solutions, RTI International, Durham, North Carolina, USA

**Keywords:** infectious pathogens, zoonoses, diagnostics, acute febrile illness

## Abstract

**Background:**

Globalization, over the past century, has extended the geographic range of infectious pathogens. Where physical distance previously protected populations from pathogens circulating in distant places, factors such as population growth, urbanization, modern transportation networks, and climate change have increased local expansion and amplification and cross-boundary spread of pathogens into naïve locations. Despite this possibility, a lack of historical endemicity of a pathogen in a region can delay its detection due to unfamiliarity with the symptomatology or limited diagnostics.

**Methods:**

We developed the One Health transmission potential framework for assessing a pathogen, using a history-agnostic approach, that incorporates—but does not rely solely on—prior detection of it, to determine its transmission potential within a region. This framework was applied to 71 pathogens for Nigeria, and the results were used to inform the selection of pathogens for the Surveillance of Acute Febrile Illness Aetiologies in Nigeria (SAFIAN) study.

**Results:**

The assessment identified transmission potential for 16 pathogens for which no previous detection in humans in Nigeria was found. The SAFIAN study screened 1200 patients for 25 pathogens with transmission potential in Nigeria. Outcomes included the detection of 11 pathogens, with no previous molecular detection in humans in Nigeria, and 9 pathogens ranked as high risk for causing Public Health Emergencies of International concern.

**Conclusions:**

These results indicate the utility of the One Health transmission potential framework and its role in global health security. To prepare for surreptitious circulation of a pathogen, a history-agnostic approach is needed, whereby evidence, beyond previous detection in a region, is used to determine pathogens' potentials.

## Teaser

This work provides a framework for assessing pathogen transmission potential within a region using a One Health framework. Its application to a surveillance study demonstrated its utility by generating unexpected findings with clinical and global public health implications.

Over the past century, global developments such as urbanization, population growth, and expansion of modern transportation networks have provided opportunities for many pathogens of public health significance to extend their reach to new, immune-naive populations. Whereas geographical constraints previously isolated most human populations and localized the reach of novel and emerging pathogens, an increasingly globalized world—connected through urban centers and transportation networks—has enabled a rapid expansion in the reach and spread of infectious diseases [1]. This is evidenced by multiple recent epidemics and a pandemic including those caused by the Zika, Ebola, and severe acute respiratory syndrome coronavirus 2 viruses [2–5].

Zoonotic spillover events can occur where there are interactions between humans or livestock and pathogen-carrying sylvatic species; such interactions can be direct (such as from bushmeat hunting [6] or animal bites [7]) or indirect (such as from exposure to animal excreta or secretions) [8–10]. These interactions are driven and mediated by a complex interplay of factors including the disturbance of previously uncultivated regions, population growth, or agricultural expansion. A newly spilled-over pathogen, if successful in replication within its new host, has an opportunity to amplify in a new population and subsequently spread. One example of this is the spillover of Nipah virus from bats into domestic pigs. This spillover was preceded by the expansion of pig farms into the bat habitats, followed by further spread through international trade routes that introduced the virus into populations in Malaysia and Singapore [11].

Climate change has been shown to alter the abiotic conditions considered to be habitable for vectors and reservoirs of human pathogens. These climate change–induced environmental changes can make regions previously unsuitable for a pathogen vulnerable to infection. An example of this is warming temperatures allowing for northern expansion of certain species of disease vectors such as *Ixodes scapularis* [12], *Aedes aegypti* and *Aedes albopictus* [13], and *Anopheles* spp [14]. These expansions were followed by the spread of pathogens in those areas such as *Babesia* spp, *Anaplasma phagocytophilum*, Powassan virus, Dengue virus, and *Plasmodium* spp [15].

Additionally, changes in human patterns of behavior also play an important role in pathogen emergence, amplification, and spread. The human population has grown significantly in the past quarter century, reaching an estimated 7.7 billion in 2022 [16]. Notably, 2010 marked the first time in human history when more humans lived in urban (compared to rural) environments [17]. This rapid urbanization and clustering of human populations in great numbers in large cities provides ideal conditions for rapid transmission of pathogens. Global or transborder spread is then achieved through international travel, human migration, and local mobility patterns, all of which have expanded significantly in recent decades [18, 19].

Diseases caused by pathogens with no prior detection in a region are not likely to be readily diagnosed. This can lead to delays or omission in detection of an outbreak or misdiagnosis of such a disease for a more common clinical illness [20–22]. Diagnostic testing options in many environments are limited, restricting testing options for pathogens that were not previously present in that location. These factors can contribute to the undetected transmission of a pathogen, including those of public health importance. Despite the known risk factors for introduction of new pathogens in any given locale, frameworks to assess a population's vulnerability (such as to inform surveillance programs) are limited. Where certain frameworks exist [23–25], the frameworks have not incorporated a focus on transmission potential, but rather on prior knowledge of a pathogen's circulation.

Pathogens each have distinct ecological niche requirements for persistence and transmission. Several factors can determine a pathogen's realized or potential niche, including its transmission pathway, required vectors or reservoirs, or the ecological conditions for its vectors or reservoirs. To determine the transmission potential of a pathogen, its transmission risk profile must be assessed for the specific region of interest (ROI). For example, inclusion of a Dengue virus in a surveillance study where there are no human cases recorded, but there is a presence of *Aedes aegypti* mosquitoes, may reveal surreptitious circulation of Dengue virus. Despite the focus on human health, abiotic and animal health are critical considerations for determining the transmission potential of a pathogen. Accordingly, the One Health framework, a multisectoral and transdisciplinary approach that recognizes that human health is closely connected to the health of animals and our shared environment, is essential to understanding and predicting the potential for a pathogen to be transmitted in a region.

To facilitate the assessment of potential for pathogen transmission, we developed a One Health assessment framework to guide the collection of information from primary scientific literature and other publicly available resources. The framework allows the user to determine the relative likelihood of detecting a pathogen in a defined location, using publicly available data related to humans, animals, insects, and the environment. This approach relies less on existing reports of a pathogen within a region and allows for the detection of pathogens circulating surreptitiously.

The One Health transmission potential framework described here informs Step 3 of a pathogen selection tool, described in Courtney et al [26]. Together, the One Health transmission potential framework and pathogen selection tool can be used to guide the selection of patients for infectious disease surveillance studies. The intended users of these tools are health researchers (eg, epidemiologists) conducting exploratory surveillance studies and whose role is to explore both immediate and future threats, including pathogens with no known previous detection within the region. The downstream results generated through use of these tools may support local Ministries of Health to prioritize their course of action and resources.

To illustrate how to apply this framework and pathogen selection tool, we conducted a case study using pathogens of global public health importance in Nigeria. The results of the One Health transmission potential framework and the pathogen selection tool [26] were used to inform the Surveillance of Acute Febrile Illness Across Nigeria (SAFIAN) study, an infectious disease surveillance study that screened specimens from 1200 acutely febrile patients for 26 pathogens. Results of this study are described here, and in detail in Courtney et al [27].

## METHODS

We developed a 5-parameter framework to assess pathogens for their transmission potential in a ROI. The approach was applied to a list of pathogens of public health importance and Nigeria was selected as the ROI.

### Compilation of a Master List of Pathogens for Assessment

A master list of pathogens of public health importance globally or locally in the Nigerian context was compiled. Courtney et al provide a comprehensive description of how this list was assembled [26]. The pathogens were primarily drawn from 2 sources: the National Institute of Allergy and Infectious Diseases, Emerging Infectious Diseases Category A and B Pathogens List [28]; and Nigeria's Integrated Disease Surveillance and Response priority list of pathogens that cause epidemic-prone diseases [29]. The master list consisted of 71 pathogens.

### Framework for Assessment

**Figure 1.**
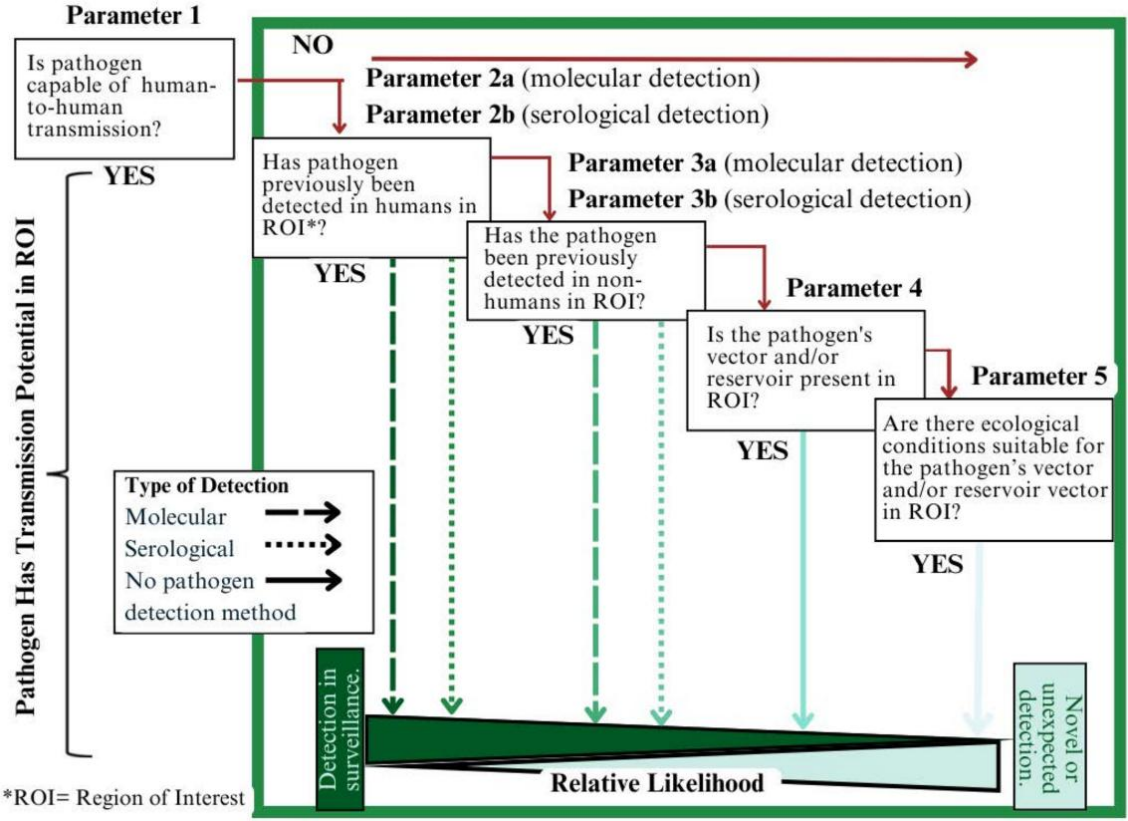
One Health transmission potential framework. Stepwise guide for assessing the transmission potential of a pathogen, within a defined region. The fulfillment of any of the 5 parameters deems a pathogen to have a transmission potential. Pathogens fulfilling parameters 2–5 (green box) are ranked on a relative scale for likelihood of detection.

We developed a 5-parameter framework for assessing transmission potential (Figure 1) and applied it to our master list of pathogens. Parameters in this framework were parameter 1: capability for human-to-human transmission; parameter 2: previous detection of the pathogen in humans in ROI; parameter 3: previous detection of the pathogen in nonhumans in ROI; parameter 4: previous detection of the pathogen's vector or reservoir in ROI; and parameter 5: ecological suitability for its vector or reservoir in the ROI. This framework is intended to be used as a stepwise tool to guide the collection of information. Once a pathogen fulfills a previous parameter in the stepwise flow, the subsequent parameters do not need to be assessed.

### Description of Parameters

#### Parameter 1: Is the Pathogen Capable of Human-to-Human Transmission?

This parameter is designed to identify pathogens that do not require an intermediate host or vector to sustain infections in humans. Pathogens that fulfill this parameter include zoonotic pathogens that spilled over into a human population and are subsequently maintained in the human population via fecal-oral, household, respiratory, sexual, or other forms of close contact transmissions among humans. These pathogens could be detected in any population or location as neither the original host nor a vector is necessary for subsequent transmission.

Modulating factors, such as sewage systems or water sources, may impact transmission likelihood; however, these considerations are outside of the scope of this framework. Assessment of this parameter involves identifying the transmission pathways for each pathogen. This information is sourced from primary scientific literature and information sheets from public health authorities (eg, Centers for Disease Control and Prevention, World Health Organization).

#### Parameter 2: Has the Pathogen Been Previously Detected in Humans in the ROI?

A pathogen that has previously been detected in humans in the ROI is a strong indicator of its potential for spread and detectability. The method of detection is also considered when estimating likelihood of detection. A pathogen detected through a molecular diagnostic test indicates an active infection, whereas a positive result from a serological assay may indicate a past infection. The former could be considered a better indicator of local infection and transmission for persons who have not recently traveled, and ranks higher for likelihood of detection on the relative likelihood scale of the One Health transmission potential framework, as compared with cases detected serologically that could be more an indicator of historical infection. Modulating factors, such as developments in infrastructure or vaccinations implemented since cases were last detected, may influence the likelihood of pathogens that fulfills this criterion; however, this is outside the scope of this framework. Assessment of this parameter includes a review of disease surveillance data for the ROI, case reports, published research findings, local newspaper articles, and reports from the local government.

#### Parameter 3: Has the Pathogen Been Previously Detected in Nonhumans in the ROI?

Zoonotic pathogens originate in animals (wildlife or livestock) or insects, before spilling over into human populations. Detection of a pathogen in insects or animals demonstrates that the ecological niche required by the pathogen is present in the ROI. Although detection does not indicate the opportunity or likelihood for a spillover event to occur, detection in a species that has significant contact with humans (eg, livestock, human-feeding mosquito species) increases the likelihood of a spillover event. Modulating factors may include a pathogen's ability to infect humans and opportunities for spillover events. Assessment of this parameter requires a review of livestock disease surveillance data and case reports, published research findings on pathogen detection in insects, wildlife, or livestock, and serological surveys or government reports. This information is also categorized by detection method (molecular or serologic).

#### Parameter 4: Has the Pathogen's Vector or Reservoir Been Detected in the ROI?

In the absence of confirmed pathogen detection, there remains a potential for transmission if a pathogen has a vector, host, or reservoir, and it is present within the ROI. Modulating factors for this to occur are cofactors for transmission and introduction of a pathogen in a population. This parameter is assessed through ecological assays in a region, research studies of realized host ranges, and mapped, gridded data of livestock or other animals.

#### Parameter 5: Are There Ecological Conditions Suitable for the Pathogen's Reservoir or Vector in the ROI?

If neither the pathogen nor its vector or reservoir has been detected previously, the ecological requirements for suitability for the vector or reservoir should be assessed. A lack of detection may indicate the lack of surveillance or efforts to identify. This parameter is assessed through climatic and ecological data and projections of potential host/vector ranges. This information can be found in the primary literature and modeling using remotely sensed data. This parameter also only applies to pathogens that have a host, reservoir, or vector.

A pathogen that meets any 1 of these 5 parameters is considered as having a detection potential in a surveillance study. The framework also includes a relative assessment for likelihood of detection, and, conversely, the likelihood for novel or unexpected detection within the ROI. Moving from left to right, for parameters 2–5, the earlier that a pathogen fulfills a parameter in the stepwise flow, the more likely that it will be detected in a surveillance study. The later that it fulfills a parameter in the stepwise flow, the less likely its detection will be.

In general, as pathogens fulfilled a parameter (moving through 1–5), the subsequent parameters were not assessed. However, biological systems are subject to variance, and strictly fulfilling a given parameter may not be the best indicator. For example, if a pathogen is capable of human-to-human transmission, but the mode of transmission does not drive or maintain outbreaks, the pathogen should be assessed for the second parameter as well. Similarly, a pathogen that fulfills the second parameter via the detection of a single case should also be assessed for the third parameter. As such, these parameters should be used as guiding steps to develop an argument for detection potential, rather than binary rules or an algorithmic decision-making tool. Information should be collected on each parameter until a parameter is fulfilled completely. Criteria for parameter fulfillment level are provided in Table 1. Finally, some pathogens have been poorly studied and basic information, such as their modes of transmission and primary vectors, is not yet known. These pathogens are not assessable using this framework.

**Table 1.** Description of Fulfillment Levels of the Parameters in the Pathogen Transmission Potential Framework

Satisfaction Level of a Parameter	Definition
Not fulfilled	The answer to the parameter's question is “no,” or information cannot be found.
Partially fulfilled	The answer to the parameter's question is “yes,” but the evidence is not strong. For example, a pathogen is capable of human-to human transmission. However, this route is not a driver of transmission; there is only one human case detected, or ecological conditions are present but only in a very small portion of the ROI.
Fulfilled	The answer to the parameter's question is “yes,” without any qualifiers or exceptions to the answer. Sufficient evidence was found that the question is fully satisfied, per the description of the parameter, rather than solely by the question posed.
Unable to assess	Parameter cannot be assessed with currently available data.

Abbreviation: ROI, region of interest.

### Description of Parameter Assessment

We assessed primary literature and other resources on each of the pathogens in the master list using parameter-specific searches in PubMed, Google Scholar, publicly available government surveillance data and reports, book chapters, and Google search engines. No date range limitation was included in the searches. Parameters were assessed in order, 1–5, per the framework (Figure 1). After a parameter was fulfilled, per the description in Table 1, no additional searches were made.

The search terms used to find information to assess each parameter were as follows:

- Parameter 1: “(*pathogen*) AND (transmission pathway) OR (transmission route) OR (transmitted to humans)”
- Parameters 2–3: “(*pathogen*) AND (*region of interest*)’
  - Subsequent review of each article to identify relevant publications that report serologic or molecular detection of pathogen in a human(s), insects(s) or animal(s).
- Parameter 4 (where applicable):
  - Compile list of vectors/reservoirs for pathogen
    - (name of vector and/or reservoir 1) OR (name of vector and/or reservoir 2) OR (name of vector and/or reservoir x) AND (region of interest)
    - For livestock: Review gridded livestock production systems maps [30].
- Parameter 5 (where applicable):
  - Compile list of ecological conditions or habitat requirements for vectors/reservoirs for pathogen.
  - Review primary literature, US Geological Survey reports, and other ecological reports for the relevant conditions, tree species, etc.
  - Access remotely sensed data (eg, via Google Earth Engine) for relevant Earth observations such as temperature, landcover data, and elevation.

Publications and resources were reviewed individually and information relevant to each parameter was extracted and sourced.

### Selection of Pathogens for Inclusion in SAFIAN Study

The results of the One Health transmission potential framework were fed into the Pathogen Selection Tool [27] to determine which pathogens to include in the SAFIAN study.

## RESULTS

We assembled a master list of 71 pathogens of global public health importance and evaluated them using the pathogen transmission potential framework. A total of 33 viruses, 26 bacteria, 2 nematodes, and 10 protozoans were included on the master list of pathogens for assessment. In total, 243 references were used across 11 data source categories (Table 2). Most of the references are found from primary scientific literature (53.5%), followed by review articles or scoping reviews (22.6%), book chapters (6.6%) and case reports/outbreak investigations (6.1%), and remotely sensed data (4.1%). The remaining categories had six or fewer references.

**Table 2.** Number of References From Each Data Source Category Used to Complete the Assessment of Transmission Potential of 71 Pathogens of Public Health Significance in Nigeria

Data Source Type	Number of References
Primary scientific literature	130
Review article/scoping review	55
Book chapters	16
Case report/outbreak investigation	15
Remotely sensed data	10
World Health Organization reports, bulletins, guidelines, or handbooks	6
US Geological Survey catalogs, International Union for Conservation of Nature threatened species lists, UN Environment Program-World Conservation Monitoring Centre, and eBird Lists	6
Food and agriculture organization assessment	2
Centers for Disease Control and Prevention Mortality and Morbidity Weekly Review	1
Local news articles	1
Federal ministry (eg, health, agriculture)	1
**Total**	243

Table 3 lists the results from the One Health transmission potential assessment for assessed pathogens, per the framework's parameters, for a subset of the pathogens assessed. This table also indicates to which level of satisfaction (Table 1) (described in Methods) that each piece of evidence fulfilled for each parameter. The complete set of results for the 71 pathogens assessed can be found in Supplementary Material 1. A total of 27 of the pathogens evaluated fulfilled parameter 1, 20 fulfilled parameter 2, 5 fulfilled parameter 3, 7 fulfilled parameter 4, and 4 fulfilled parameter 5. Six pathogens were determined to not have a transmission potential in Nigeria. Two pathogens, the Chapare and Lujo viruses, did not fulfill any of the 5 parameters because they were deemed not assessable by this framework (Table 4). These viruses are both newly discovered New World arenaviruses, which have no known vector, reservoir, or host. There have been too few cases for a valid ecological niche model to be developed, based on the locations of their outbreaks.

**Table 3.** **Transmission Potential Framework Assessment Results for Pathogens of Public Health Significance in Nigeria** ^a^

Pathogen	Evidence For Satisfaction of Each Transmission Potential Framework Parameter
Rubeola virus	**Parameter 1:** Highly contagious and transmitted from human to human through the respiratory route [31]. **(Parameter 1 fulfilled)**
Dengue virus	**Parameter 1:** Vector-borne disease transmitted to humans by *Aedes aegypti* and *Aedes albopictus* mosquitoes [32]. **(Parameter 1 unfulfilled)** **Parameter 2:** Dengue and other arboviruses are widespread throughout Nigeria [33–35]. (**Parameter 2a fulfilled)**
*Rickettsia* spp.	**Parameter 1:** Transmission of rickettsiae to humans is primarily via the bite of infected arthropods such as ticks, fleas, mites, and lice. Infection is also possible through inhalation of airborne bacteria (in *Rickettsia prowazekii)*, or when bacteria present in arthropod feces enter the body through bite wounds, the eyes, or mucous membranes (in *Rickettsia typhi)* [36]. **(Parameter 1 unfulfilled)** **Parameter 2:** No documented human cases of any *Rickettsia* spp. in Nigeria [37]. **(Parameter 2 unfulfilled)** **Parameter 3:** Molecular detection in dogs and ticks [38] and ticks from the vegetation and livestock in Nigeria [39]. **(Parameter 3a fulfilled)**
Hantavirus	**Parameter 1:** Transmitted to humans via inhalation of aerosolized excreta or secreta from infected rodents, with some suspicion but no real evidence of human-to-human transmission [40]. **(Parameter 1 unfulfilled)** **Parameters 2–3:** Serological evidence in humans [41] and molecular evidence in rodents in Western Africa [42–44]. **(Parameter 2 and 3 partially fulfilled)** **Parameter 4:** Various African shrews have been shown to be the zoonotic hosts in countries throughout Africa including *Crocidura theresae* [42] and *Crocidura douceti* [43] in Guinea and *Crocidura obscurior* in the Ivory Coast [44]. Although Nigeria appears to have the geographic boundary for *Crocidura* spp. expanding inland, parts of Nigeria are inhabited by multiple *Crocidura* taxa [45]. **(Parameter 4 fulfilled)**
*Yersinia pestis* (plague)	**Parameter 1:** Carried by rodents and transmitted within them through a flea vector (*Xenopsylla cheopis*) [46, 47]. **(Parameter 1 unfulfilled)** **Parameters 2–3:** No human or animal cases detected [48]. **(Parameters 2–3 unfulfilled)** **Parameter 4:** Found in small numbers 30+ y ago [49] but not detected since [50]. **(Parameter 4 unfulfilled)** **Parameter 5:** Potential distribution of plague in Nigeria-based ecological niche modeling of environmental covariates including potential evapotranspiration, normalized difference in vegetation index of various y, and minimum and maximum temperatures of the coldest and hottest months, respectively [51]. **(Parameter 5 fulfilled)**

^a^This table contains the findings for a subset of pathogens. The remaining findings can be found in Supplementary Material 1.

**Table 4.** Summary of Literature Review Findings for 71 Pathogens, Evaluated Using One Health Transmission Potential Framework

Transmission Potential Framework Parameter	Pathogens Fulfilled at Each Parameter	Relative Likelihood of Detection
**Parameter 1** ^a^ Is the pathogen capable of human-to-human transmission?	*Bordetella pertussis* *Burkholderia pseudomallei* (melioidosis) Caliciviruses *Clostridium botulinum* *Corynebacterium diphtheriae* *Cryptosporidium parvum* *Cyclospora cayetanensis* Diarrheagenic *E. coli* Ebola virus *Entamoeba histolytica* *Giardia lamblia* Hepatitis A virus Hepatitis E virus *Listeria monocytogenes* *Microsporidia* Monkeypox virus *Mycobacterium tuberculosis* *Naegleria fowleri* *Neisseria meningitidis* Pan-orthopoxvirus Poliovirus Rubeola virus Rubella virus Severe acute respiratory syndrome coronavirus-2 *Shigella* spp. *Streptococcus pneumoniae* *Vibrio cholerae*	Not applicable. Pathogens that fulfill parameter 1 are not ranked on the scale for relative likelihood of detection.
**Parameter 2** Has the pathogen been previously detected in humans in the ROI?	Parameter 2a: Molecular Detection *Brucella* spp. *Campylobacter jejuni* *Dracunculus medinensis* (diagnosed by direct examination) Lassa virus *Leishmania* spp. *Leptospira* spp. *Mycobacterium ulcerans* Pan-Salmonella *Plasmodium* spp. Rabies virus *Toxoplasma gondii* *Trypanosoma brucei* *Wuchereria bancrofti*, *Brugia malayi*, and *Brugia timori* Yellow fever virus	…
	Parameter 2b: Serological Detection Crimean-Congo hemorrhagic fever virus Chikungunya virus *Coxiella burnetii* Rift Valley fever virus West Nile virus Zika virus	
**Parameter 3** Has the pathogen been previously detected in non-humans in the ROI?	Parameter 3a: Molecular Detection • *Bartonella* spp. • Dengue virus • *Rickettsia* spp.: (*Rickettsia conorii israelensis, Rickettsia africae. Rickettsia aeschlimannii, R. aeschlimannii*, and *R. annulatus*) • *Yersinia enterocolitica* Parameter 3b: Serological Detection • Japanese encephalitis virus	
**Parameter 4** Is the pathogen's vector or reservoir present in the ROI?	*Burkholderia mallei* (glanders) *Chlamydia psittaci* (Psittacosis) *Francisella tularensis* Hantavirus LaCrosse encephalitis virus O’nyong’nyong virus St. Louis encephalitis virus	
**Parameter 5** Are there ecological conditions suitable for the pathogen's vector or reservoir in the ROI?	*Bacillus anthracis* Marburg virus *Orientia tsutsugamushi* *Yersinia pestis*	
**No transmission potential**	*Balamuthia mandrillari* Eastern equine encephalitis virus Guanarito virus Junin virus Machupo virus Nipah virus (Parameter 5 partially fulfilled) Venezuelan equine encephalitis virus (Parameter 5 partially fulfilled)	…
**Unable to Assess**	Chapare virus Lujo virus	…

Abbreviations: *E. coli*, *Escherichia coli*; ROI, region of interest.

^a^Pathogens that fulfilled parameter 1 were not ranked on the relative likelihood of detection scale, which ranks the available evidence for detection in humans from most to least likely based on the quality and strength of evidence available for transmission potential. Pathogens capable of human-to-human transmission, not requiring an intermediary host or vector, are not ranked on this scale.

**Figure 2.**
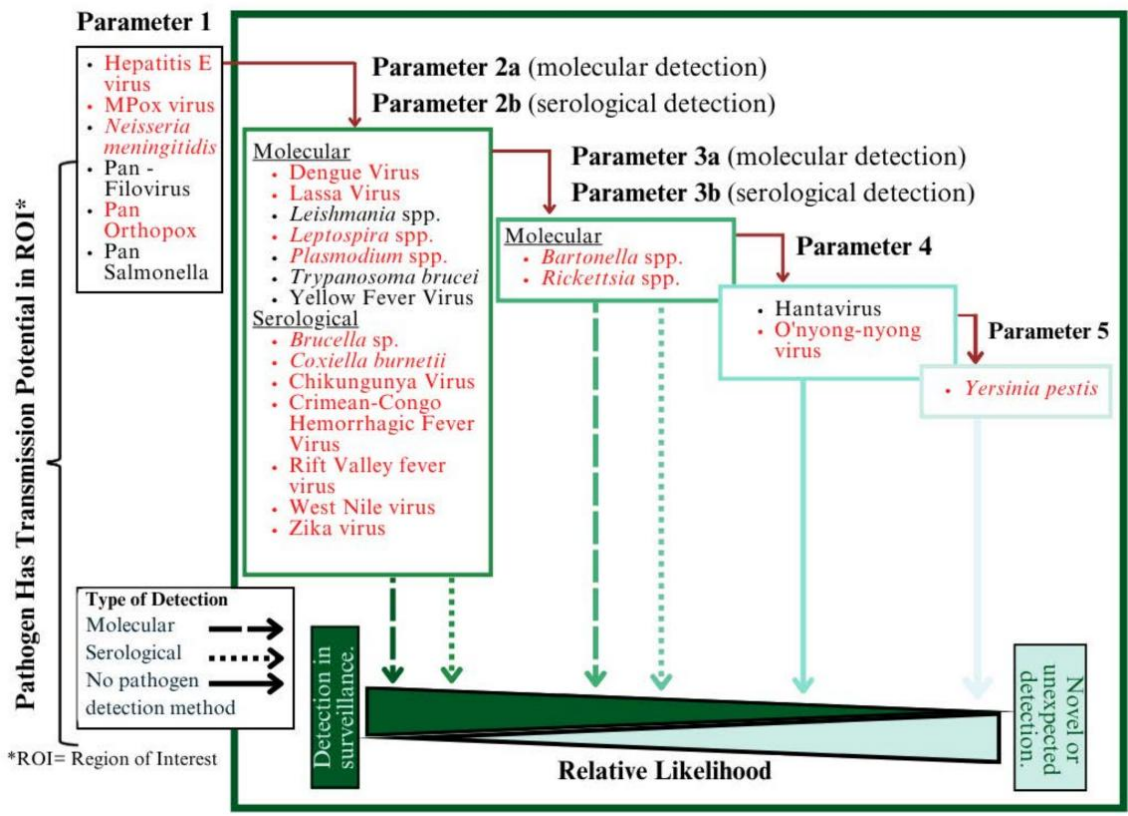
SAFIAN results, per the One Health transmission potential framework. Each pathogen that was screened for in the Surveillance of Acute Febrile Illness Aetiologies in Nigeria (SAFIAN) study is mapped to the One Health transmission potential framework parameter it fulfilled before the SAFIAN study. All pathogens detected in the SAFIAN study now fulfill parameter 2a of the One Health transmission potential framework, as SAFIAN used a molecular technique to screen human specimens, which fulfilled the criteria for parameter 2a. Pathogens listed in red were detected in at least 1 SAFIAN participant. The pathogens in black were not detected in any SAFIAN participants.

The results of this assessment were used to inform step 3 of the pathogen selection tool [26], and a final list of 25 pathogens were selected for inclusion in the SAFIAN study. The results of the SAFIAN study are presented in Figure 2. Each pathogen selected for inclusion is overlaid with the One Health transmission potential framework parameter it fulfilled at the time that it was assessed (before the SAFIAN study). A total of 19 of the pathogens selected for inclusion in SAFIAN were detected in at least 1 participant. All pathogens detected in the SAFIAN study now fulfill parameter 2a of the One Health transmission potential framework, as SAFIAN used a molecular technique to screen human specimens, which fulfills the criteria for parameter 2a.

There were 4 pathogens detected in the SAFIAN study that, to the best of our knowledge, represent the first molecular detection in humans in Nigeria [27] (those which fulfilled parameters 3–5). Two of these pathogens had previously been detected in animals or insects, but not humans (parameter 3: *Rickettsia* spp. and *Bartonella* spp.) and 2 had never been detected in Nigeria (parameter 4: O’yong’nyong virus; parameter 5: *Yersinia pestis*), per assessment using the One Health transmission potential framework.

The 4 pathogens that fulfilled parameters 3–5, for which no record of human infection in Nigeria was found before SAFIAN, were ranked by the One Health transmission potential framework assessment as unlikely to be detected. Despite that, at least 1 of these 4 pathogens was detected in more than one quarter (26.8%) of the AFI population screened in the SAFIAN study. Despite the lack of prior detections in humans in Nigeria, *Rickettsia* spp. was detected in 312 SAFIAN participants. *Plasmodium* spp., a pathogen with known endemicity in Nigeria and a target of surveillance and intervention campaigns, was only detected in 293 participants [27].

The SAFIAN study also detected multiple high-consequence pathogens. Nine of the pathogens detected—pan-orthopox, monkeypox virus, dengue virus, Lassa fever virus, Crimean-Congo hemorrhagic fever virus, chikungunya virus, Zika virus, Rift Valley Fever virus, and *Yersinia pestis*—are ranked as high risk for causing Public Health Emergencies of International concern, according to the 2024 World Health Organization Pathogen Prioritization list [52].

Two of these pathogens, *Rickettsia* spp. and Chikungunya virus, had not been included in any Nigerian surveillance study that we found. The other 9 pathogens detected—Crimean-Congo hemorrhagic fever, Rift Valley fever virus, *Yersinia pestis*, *Neisseria meningitidis*, O'nyong’nyong virus, hepatitis E virus, *Brucella* spp., *Bartonella* spp., and *Coxiella burnetii*—were included in a surveillance study conducted by the Nigerian Centers for Disease Control and Prevention (2022–2023); however, the results of that study have not been made public. The list was shared via personal communications.

## DISCUSSION

Our goal was to develop a tool to assess a region's vulnerability to a list of pathogens, even when direct measurements were not available, by assessing their transmission there. A total of 71 pathogens were assessed for their transmission potential in Nigeria. The results of this assessment were used to inform step 3 of a pathogen selection tool [26] and the results informed which pathogens to surveil in an acutely febrile population for the SAFIAN study. The history-agnostic approach led to the inclusion and detection of 4 pathogens for which there was no previous public record of human cases in Nigeria and 9 pathogens ranked as high risk for causing Public Health Emergencies of International concern, according to the 2024 World Health Organization Pathogen Prioritization list [52] including those that are currently not under surveillance by local health authorities or tested for by local clinicians.

The number of pathogens detected in the SAFIAN study that were determined to have a transmission potential, but unlikely to be detected, and the number of high-risk pathogens detected that were not previously included or reported on in other surveillance studies, demonstrates the utility of the methodologies developed to select pathogens for surveillance studies and highlights several important concepts for infectious disease surveillance: (1) The need for ongoing infectious disease surveillance studies. Health researchers have the responsibility of exploring both immediate and potential future threats to assist governments and Ministries of Health to prioritize their course of action and resources. This must include consideration for pathogens with no known previous detection within the region. (2) The detection of these pathogens in a surveillance study using a research use–only tool does not provide information for clinical staff. These findings should contribute to a pipeline for confirmatory testing and follow-up with patients that can provide actionable information to appropriately treat patients with AFI and to prevent, detect, and respond to pandemic threats prior to adverse impacts on global health security. (3) The development and use of the One Health–informed approach is essential to understanding disease distribution and occurrence. Based on human cases alone, we would not have considered including four of these pathogens in the SAFIAN study—*Bartonella* spp., O’nyong’nyong virus, *Yersinia pestis,* and *Rickettsia* spp. These pathogens fulfilled parameters 4 or 5 of the One Health transmission potential framework. Moreover, the methods employed selected for inclusion of 11 pathogens, with no documented incidence in humans in Nigeria, and were outside of the scope of published surveillance studies there. Accordingly, the use of this One Health framework identified significant public health threats with clinical implications that were not previously known.

The One Health transmission potential framework presented here complements the efforts of the One Health zoonotic disease prioritization tool used in Nigeria, Kenya, and Vietnam [23–25], which relies heavily on local expertise and existing records of a disease, combined with country-specific priorities. In comparison, the One Health transmission potential framework uses an agnostic approach and focuses heavily on niche and transmission potential of a pathogen rather than previous detection. This tool works despite the paucity of critical data commonly used for disease prioritization and surveillance efforts. The framework described here is designed to assess pathogens that could be in circulation in a region, despite no previous detections and no known public health burden there. Accordingly, this framework is intended for researchers conducting surveillance studies. Other tools, such as the One Health zoonotic disease tool, are better designed for governments who need to decide how best to use limited resources. This framework approach is needed in an ever-connected world, where distance from a place of spillover or transmission no longer confers the same protection from a pathogen. Furthermore, surveillance of pathogens that are not currently known to be present in a region may contribute to global biological threat reduction via early detection and subsequent efficient public health response.

This One Health transmission potential framework and pathogen selection tool [26] are applicable to any list of pathogens and any defined location. The framework offers an accessible and low-resource approach to assessing transmission potential for any pathogen in any ROI. By using exclusively publicly available data sources, the framework is time and cost accessible.

Limitations of this work include lack of geographic granularity. For the purposes of this assessment, we considered information found in any location in Nigeria to be representative of the entire country, despite significant heterogeneity in ecological conditions that influence transmission potential. Another limitation to the work is that Nigeria may be better resourced than other African countries with regards to the number of academic institutions and researchers. This may equate to more data availability as compared with other countries. With regards to the paucity of data in an ROI, more focus should be given to remotely sensed data and ecological prediction models, which require fewer locale-specific data inputs. Finally, the framework assesses transmission potential based strictly on its core transmission requirements and does not attempt to assess modulating factors such as sewage and waste management, water purification processes, vaccination availability/rates, and air filtration systems, all of which can be important predictors of disease occurrence.

The findings of this work could be used to inform public health and medical practice, improving health outcomes as well as contributing to early warning for outbreaks. The results demonstrate the need to look beyond direct measurements of human cases and to consider what could be in circulation within a population rather than what is currently known to be circulating through a One Health lens that supports this assessment. This proactive approach is needed in an ever-connected and globalized world that is vulnerable to infectious threats.

## Supplementary Material

ciaf487_Supplementary_Data

## Supplementary Data

Supplementary materials are available at Clinical Infectious Diseases online. Consisting of data provided by the authors to benefit the reader, the posted materials are not copyedited and are the sole responsibility of the authors, so questions or comments should be addressed to the corresponding author.
