# Supplementary material for: Design and Implementation of a One Health Framework for Evaluating the Transmission Potential of Pathogens of Public Health Interest for Surveillance Studies
Source: Clin Infect Dis. 2025 Nov 20;81(Suppl 4):S202–12. doi: 10.1093/cid/ciaf487 (PMC12631772; doi:10.1093/cid/ciaf487)
Supplement: ciaf487_Supplementary_Data [file ciaf487_supplementary_data.docx]

Supplemental Material 1

# Complete Literature Review Findings for Detection Potential in Nigeria, for Pathogens of Public Health Significance

| **Pathogen** | **Evidence for Detection** |
| --- | --- |
| **Viral Pathogens** | |
| Caliciviruses | **Parameter 1:**  Typically transmitted between humans through fecal contact. (1) Transmission can also occur via the ingestion of contaminated food or water. (2) **(Parameter 1 fulfilled)** |
| Chapare virus | **Parameter 1:**  Human-to-human transmission has not been document for Chapare, but it has been for other Mammarenaviruses.(3,4) **(Parameter 1 unfulfilled)**  **Parameter 2:**  Only documented cases to date have been in Bolivia,(5–7) discovered in 2023. **(Parameter 2 unfulfilled)**  Transmission to humans is likely through exposure to infected rodents or their excreta.(4,5)  **Parameters 3-5:**  Reservoir not yet identified.(8) **(Parameters 3-5 not assessable)** |
| Chikungunya virus (CHIKV) | **Parameter 1:**  No human-to-human transmission documented. Principle vector to humans is *Aedes aegypti* mosquitoes.(9) **(Parameter 1 unfulfilled)**  In a 2020 meta-analysis of CHIKV infection in Nigeria, pooled results showed 3.8% (95% CI: 2.0-6.4) CHIKV RNA-positive cases.(10) **(Parameter 1 partially fulfilled)**  **Parameter 2:**  In a 2020 meta-analysis of CHIKV infection in Nigeria, pooled anti-CHIKV IgM and IgG seroprevalences were 26.7% (95% CI: 23.2 - 30.4) and 29.3% (95% CI: 26.2 -32.6), respectively.(10) In Kogi state in Nigeria, where CHIKV had never been previously diagnosed, 243 acute febrile illness patients who tested negative for malaria and typhoid were screened for CHIKV serology; 25% demonstrated having had a previous infection of CHIKV.(11) **(Parameter 2b fulfilled)** |
| Crimean-Congo hemorrhagic fever virus | **Parameter 1:**  The most common and efficient route of infection is through infected tick bites. Human-to-human transmission can occur through direct exposure to blood or secretions; however, this is not thought to be a driver for outbreaks.(12) **(Parameter 1 partially fulfilled)**  **Parameter 2:**  One human case previously identified in Nigeria through a molecular diagnostic test of an undiagnosed acute febrile patient captured in a study that collected specimens from malaria and typhoid negative acutely febrile patients from 2010 to 2014.(13) **(Parameter 2a partially fulfilled)**  Serosurveys in Nigeria reveal an overall seroprevalence in humans of 2.4%–10.6%.(13,14) **(Parameter 2b fulfilled)**  **Parameter 3:**  Studies have revealed an overall seroprevalence of ~25% among cattle at sites distributed throughout Nigeria.(15) **(Parameter 3b fulfilled)** |
| Dengue virus (DENV) | **Parameter 1:**  Vector-borne disease transmitted to humans by *Aedes aegypti* and *Aedes albopictus* mosquitoes.(9) **(Parameter 1 unfulfilled)**  **Parameter 2:**  DENV and other arboviruses are widespread throughout Nigeria.(16–18) (**Parameter 2a fulfilled)** |
| Eastern equine encephalitis virus (EEE) | **Parameter 1:**  Transmitted to humans via infected mosquito bite. The following mosquito species, which feed on both avian (reservoir) and mammalian hosts, are *Culiseta melanura* and *Coquillettidia perturbans.*(19) **(Parameter 1 unfulfilled)**  **Parameters 2-3:**  EEE has only been detected in the Americas.(20) **(Parameters 2-3 unfulfilled)**  **Parameter 4:**  EEE is transmitted to humans via *Culiseta melanura* and *Cs. morsitans* mosquitoes. *Cs. Morsitans’* known geographic distribution is throughout northern United States, through Canadian Yukon Territory into Alaska. They have been found as far south as Delaware. They were once recorded in northwestern Spain.(21) *Culiseta melanura* has a wider geographic distribution and ranges from southern Quebec, Canada, to south Florida. They can be found spanning the United States from the eastern border of the Great Plains to the Atlantic coast. They have also been found in the Caribbean.(22) **(Parameter 4 unfulfilled)**  **Parameter 5:**  *Cs. morsitans* (23) *and Cs. melanura* (24) habitat is semipermanent woodland swamps. There has been minimal classification of the swamps and tree species in Nigeria. Nigeria is home to mangrove swamps, which are not hardwood. The Otuwe forest of the Nigeria Delta is a freshwater swamp. One study classified the tree species of this forest and three of four most abundant tree species, Diospyros mesipiliformis, Sterculia oblonga, and Sterculia rhinopetala, are hardwood species.(23) However, laboratory experiments have shown that *Cs.* *morsitans*/*fumipennis* avoid temperatures above 30°C, and temperatures in Ondo state are consistently at or above 30°C.(25) **(Parameter 5 unfulfilled)** |
| Ebola virus (EBOV) | **Parameter 1:**  Human-to-human transmission through close contact with blood or body fluids(26) including exposure to infected semen during sexual contact.(27) **(Parameter 1 fulfilled)** |
| Guanarito virus | **Parameter 1:**  Transmitted to humans from contact with excreta of wild rodents.(28) One documented case with suspected human-to-human transmission.(29) **(Parameter 1 partially fulfilled)**  **Parameters 2-3:**  Only human case to date has been documented in Venezuela.(28,30) **(Parameters 2-3 unfulfilled)**  **Parameter 4:**  No surveys for virus in Nigeria found. **(Parameter 4 unfulfilled)**  **Parameter 5:**  Reservoirs thought to be indigenous rodents, Zygodontomys brevicauda,(28,30) which are found in eastern Central America and northern South America, and Costa Rica, south to western Ecuador, east to French Guiana, and on the islands of Trinidad and Tobago. Characteristics of the geographic range of Z. brecicauda include closed-canopy forests.(31,32) Most collection records are from elevations below 100m, (33) which are present in Nigeria (34,35); however, it does not have closed canopy forest.(36) **(Parameter 5 unfulfilled)** |
| Hantavirus | **Parameter 1:**  Transmitted to humans via inhalation of aerosolized excreta or secreta from infected rodents, with some suspicion but no real evidence of human-to-human transmission.(37) **(Parameter 1 unfulfilled)**  **Parameters 2–3:**  Serological evidence in humans(38) and molecular evidence in rodents in Western Africa.(39–41) **(Parameters 2-3 partially fulfilled).**  **Parameter 4:**  Various African shrews have been shown to be the zoonotic hosts in countries throughout Africa including Crocidura theresae (39) and Crocidura douceti (40) in Guinea, and Crocidura obscurior in the Ivory Coast.(41) Although Nigeria appears to have the geographic boundary for Crocidura spp. expanding inland, parts of Nigeria are inhabited by multiple Crocidura taxa.(42) **(Parameter 4 fulfilled)** |
| Hepatitis A virus | **Parameter 1:**  Human-to-human transmission is common and occurs primarily via the fecal-oral route through close personal contact or consumption of contaminated food or water.(43) **(Parameter 1 fulfilled)** |
| Hepatitis E virus | **Parameter 1:**  Transmitted primarily by the fecal–oral route or through contaminated water.(44) It can also be transmitted across species between humans, pigs, boars, deer, chickens, and rabbits.(45–47)  **(Parameter 1 fulfilled)** |
| Japanese encephalitis virus (JEV) | **Parameter 1:**  JEV is transmitted to humans through the bite of infected mosquitoes, primarily *Culex* species, particularly *Culex tritaeniorhynchus*.(48) **(Parameter 1 unfulfilled)**  **Parameter 2**:  JEV is found in 25 countries across eastern and southeastern Asia. Its detection has not been reported in humans or animals in Nigeria.(49) **(Parameter 2 unfulfilled)**  **Parameter 3**:  Detected serologically in pigs in southwestern Nigeria.(50) **(Parameter 3b fulfilled)** |
| Junin virus | **Parameter 1:**  Transmitted to humans through the disturbance of the habitat of its reservoirs, *Calomys musculinus* and *Calomys laucha*, which inhabit grain fields. Infection occurs through cuts and abrasions or through aerosolized dust from rodents caught in harvesting machinery.(51) **(Parameter 1 unfulfilled)**  **Parameters 2-3:**  Endemic in the humid pampas of Argentina. No human or animal cases detected in Nigeria.(52) **(Parameters 2-3 unfulfilled)**  **Parameter 4:**  The known geographic distribution of *Calomys musculinus* is limited to Northern Argentina; however, range expansion has been documented to new regions within Argentina.(53)  The known geographic distribution of *Calomys laucha* is limited to the South American countries of Bolivia, Paraguay, Argentina, Uruguay, and Brazil.(54) **(Parameter 4 unfulfilled)**  **Parameter 5**:  *C. musculinus* occupies a wide variety of habitats, including natural grasslands, shrub steppes, crop field borders, and human-disturbed environments, such as wastelands, railroads, and urban garbage dumps.(55,56) Species distribution modeling across Argentina and surrounding countries, using climate change predictions showed an expansion of a suitable habitat for this species to beyond Argentina’s borders.(52) In addition to human-disturbed environments conducive to the habitat of *C. musculinus* (e.g. urban garbage dumps and railroads), Nigeria is also home to cultivated and natural/seminatural grasslands*.*(57,58)*;* however, this species prefers yearly average temperatures between 10° and 17°C, and annual temperature ranges of 22°–27°C(52) ,which is much cooler than average Nigerian temperatures*.*(25) **(Parameter 5 unfulfilled)** |
| LaCrosse encephalitis (LACV) | **Parameter 1:**  La Crosse virus is transmitted to humans through the bite of infected mosquitoes, primarily *Aedes triseriatus.*(59) *Aedes albopictus*and*Aedes japonicus* have recently been identified as competent vectors for transmission of La Crosse virus and are likely playing an emerging role in the maintenance and spread of the virus to new areas.(60) There are no documented cases of human-to-human spread contact. **(Parameter 1 unfulfilled)**  **Parameters 2-3:**  Only documented cases of LACV in North America.(61) (**Parameters 2-3 unfulfilled)**  **Parameter 4:**  The known geographic range of *Aedes triseriatus* is limited to North America. They can be found southern Canada and eastern United States. However, *Aedes albopictus* mosquitoes have been identified across sites in Nigeria.(62) (**Parameter 4 fulfilled)** |
| Lassa virus | **Parameter 1:**  Transmission from rodents to humans occurs through direct exposure to rodent fluids such as urine, saliva, and blood or indirect exposure via surfaces and foodstuffs contaminated by these fluids.(63,64) No significant human-to-human transmission recorded to date. **(Parameter 1 unfulfilled)**  **Parameter 2:**  Significant number of human cases recorded each year.(65,66) **(Parameter 2a fulfilled)** |
| Lujo virus (LUJV) | **Parameter 1:**  Nosocomial transmission has been recorded in some outbreaks.(67–69) **(Parameter 1 partially fulfilled)**  **Parameters 2-3:**  No previous detection of LUJV in Nigeria or in Western Africa in humans or in animals.(67) **(Parameters 2-3 unfulfilled)**  **Parameters 4-5:**  Unknown ecology, distribution, and mode of transmission from reservoir host to humans and distribution and prevalence of LUJV in humans and rodents.(68) **(Parameters 4-5 not assessable)** |
| Machupo virus | **Parameter 1:**  Human-to-human transmission has been recorded;(70,71) however, it is not thought to be a driver of transmission. Calomys callosus rodents are its vector.(70) **(Parameter 1 partially fulfilled)**  **Parameters 2–3:**  Only cases detected, in both humans and animals, were in South America.(72) **(Parameters 2-3 unfulfilled)**  **Parameter 4:**  Reservoir is a large vesper mouse found in Argentina, Bolivia, Brazil, and Paraguay.(73) **(Parameter 4 unfulfilled)**  **Parameter 5:**  Reservoir appears to demonstrate nidality (74) owing to host genetics at the clade level (75,76) making it unlikely to find vector-competent species outside of the area that they currently inhabit. **(Parameter 5 unfulfilled)** |
| Marburg virus | **Parameter 1:**  Human-to-human transmission is facilitated by direct contact with blood or body fluids of infected individuals occurs. Household and health care spread is common. Outbreaks are usually initiated with a spillover event.(77–79) **(Parameter 1 partially fulfilled)**  **Parameters 2-3:**  First case in Western Africa in 2021, did not have a history of travel.(80) No known cases in humans or animals in Nigeria. **(Parameters 2–3 unfulfilled)**  **Parameter 4:**  Host presumed to be the Egyptian fruit bat (*Rousettus aegyptiacus*),(81) which has a geographic distribution along the western coast of Africa, including parts of Nigeria.(82) **(Parameter 4 partially fulfilled)**  **Parameter 5:**  Ecologic niche modeling predicted environmental suitability for Marburg viruses in parts of southern Nigeria, based on a number of environmental covariates present at previous outbreaks, including range and mean values of enhanced vegetation index and land surface temperature (day and night), elevation, and potential evapotranspiration.(83) **(Parameter 5 fulfilled)** |
| Monkeypox virus | **Parameter 1**:  Routes of transmission to humans include percutaneous exposure, such as through direct exposure of skin, especially broken skin; direct exposure of mucous membranes, such as those found in the mouth, vagina, and rectum; and inhalation of infectious particles into the respiratory tract.(84,85) **(Parameter 1 fulfilled)** |
| Nipah virus | **Parameter 1:**  Transmission to humans via the fruit bat, *Pteropus medius.*(86) Human-to-human transmission can also occur through contact with bodily fluids.(87) **(Parameter 1 partially fulfilled)**  **Parameters 2-3:**  No molecular or serological detection in humans or animals in Nigeria.(88) **(Parameters 2-3 unfulfilled)**  **Parameter 4:**  The geographic range of *Pteropus medius* extends from Pakistan in the west and north through most of India and Bangladesh and through Nepal to Myanmar in the east and Maldives in the south.(89) **(Parameter 4 unfulfilled)**  **Parameter 5:**  *P. medius* roosts in 35+ tree species, which represent various biogeographic zones and tree species, which are also different in their general physiognomy and habit. Accordingly, the bat vector for Nipah virus is adept to a wide variety of tree species for roosting, which span distributional range.(90) **(Parameter 5 partially fulfilled)** |
| O’nyong-nyong virus (ONNV) | **Parameter 1:**  Transmitted to humans via infected mosquito bite and is primarily transmitted through the bite of *Anopheles funestus* and *Anopheles gambiae* mosquitoes.(91) *Anopheles* *stephensi,* the invasive mosquito species of Asian origin, now present in the Horn of Africa and spreading east, is also vector competent for ONNV in a laboratory setting.(92) **(Parameter 1 unfulfilled)**  **Parameters 2-3:**  No human or animal cases detected in Nigeria.(93) **(Parameters 2-3 unfulfilled)**  **Parameter 4:**  Various *Anopheles* spp. mosquitoes are present and distributed throughout Nigeria.(94) **(Parameter 4 fulfilled)** |
| Pan-Orthopoxvirus | **Parameter 1:**  Outbreaks caused by viruses in the Orthopox genus, including Variola virus, Vaccina virus, Monkey pox, and Buffalopox virus, typically begin with animal-to-human transmission, which can then lead to human-to-human transmission.(95) **(Parameter 1 fulfilled)** |
| Poliovirus | **Parameter 1**:  Fecal-oral transmission to humans (no intermediate host required).(96) **(Parameter 1 fulfilled)**  **Parameter 2:**  Has been endemic in Nigeria in past years,(97) although there has not been any documented case for the last 3 years.(98) **(Parameter 2 fulfilled)** |
| Rabies virus | **Parameter 1**:  Transmitted to humans through the saliva of an infected host, namely from bites from domesticated dogs(99)^,^(100) and through contact with other bodily fluids, as a result of slaughtering practices.(101) **(Parameter 1 unfulfilled).**  **Parameter 2:**  Documented human cases in Nigeria and remains a significant public health threat there in both humans and dogs.(102,103) *Note: Cases detected through antigen tests.* **(Parameter 2a fulfilled)** |
| Rift Valley fever virus | **Parameter 1**:  Transmitted to humans through mosquitoes of the *Aedes* and *Culex* genera.(104–106) **(Parameter 1 unfulfilled)**  **Parameter 2:**  No molecular detection to date.(107) Serological detection in humans(108) and in animals.(109,110) **(Parameter 2b fulfilled)** |
| Rubella virus | **Parameter 1**:  Human-to-human transmission through respiratory route and transmitted congenitally.(111) **(Parameter 1 fulfilled)** |
| Rubeola virus (Measles Disease) | **Parameter 1**:  Highly contagious and transmitted from human to human through the respiratory route.(112) **(Parameter 1 fulfilled)**  **Parameter 2**:  Is endemic in Nigeria(113) and affects mainly unvaccinated children. **(Parameter 2a fulfilled).** *Per WHO case definition, serological detection classifies an active case.* |
| Severe Acute Respiratory Syndrome Coronavirus 2 | **Parameter 1**:  Humans are infected through contact, droplet, airborne, fomite, fecal-oral, bloodborne, mother-to-child, and animal-to-human transmission.(114) **(Parameter 1 fulfilled)** |
| St. Louis encephalitis virus (SLEV) | **Parameter 1:**  Transmission to humans occurs primarily through bites from infected *Culex* mosquitoes(115) including *Cx. pipiens* and *Cx. quinquefasciatus*, *Cx. tarsalis* and *Cx. nigripalpus*. **(Parameter 1 unfulfilled)**  **Parameters 2-3:**  Reported cases are limited to the Americas.(116) **(Parameters 2-3 unfulfilled)**  **Parameter 4:**  *Cx. pipiens*(117) and *Cx. quinquefasciatus* (118) are both found in Nigeria. **(Parameter 4 fulfilled)** |
| Venezuelan equine encephalitis virus (VEEV) | **Parameter 1:**  Transmitted to humans via infected mosquito bite. VEE uses multiple different mosquito vectors; however, the drivers of outbreaks appear to be driven by *Aedes taeniorhynchus* in coastal areas. Enzootic VEEV strains is transmitted by mosquitoes in the Spissipes section of the *Culex* (*Melanoconion*) subgenus.(119) **(Parameter 1 unfulfilled)**  **Parameters 2-3:**  Outbreaks have not been reported outside of the Americas.(120) **(Parameters 2-3 unfulfilled)**  **Parameter 4:**   The subgenus *Melanoconion* typically resides in the Neotropical region. *Aedes taeniorhynchus* are limited to Mexico and the southern United States.(119) **(Parameter 4 unfulfilled)**  **Parameter 5:**  The habitat of *Aedes taeniorhynchus* is typically coastal. It breeds in saltmarshes flooded by tides or rains. They can also be found in fresh water and in inland brackish water swamps.(121) There are no salt marshes in Nigeria. (122) The eco-vegetation zones, such as brackish water swamps, are not well documented across Nigeria; however, one publication mentioned two local government areas that have brackish water swamps.(123) **(Parameter 5 partially fulfilled)** |
| West Nile virus | **Parameter 1:**  West Nile virus is primarily transmitted to humans through bites from infected Culicidae mosquitoes.(124) However, there have also been occasional cases of transmission between humans through organ transplantation, blood product transfusions, trans-placental, and breast-feeding transmission routes. (125) **(Parameter 1 partially fulfilled)**  **Parameter 2:**  Serological evidence of WVN detected in humans and animals throughout Nigeria.(126) **(Parameter 2b fulfilled)** |
| Yellow fever virus | **Parameter 1**:  No human-to-human transmission documented. Transmission requires a mosquito vector.(127) **(Parameter 1 unfulfilled)**  **Parameter 2:**  Documented human cases in Nigeria.(128–130) **(Parameter 2a fulfilled)** |
| Zika virus | **Parameter 1:**  Transmission of Zika virus in humans occurs mainly through bites from infected *Aedes aegypti* mosquitoes.(131) However, sexual transmission, transmission from mother to fetus, and blood transfusion have also been documented.(132) **(Parameter 1 partially fulfilled)**  **Parameter 2:**  Was reported in 1954 (133) and then again in 1975 (134) among AFI patients. More recently, ZIKV was detected serologically in pregnant women (2016). (135) **(Parameter 2b fulfilled)** |
| **Bacterial Pathogens** | |
| *Bacillus anthracis* (Anthrax) | **Parameter 1**:  No human-to-human transmission documented. Human disease derives from contact with livestock or animal products and through inhalation of spores from soil.(136) **(Parameter 1 unfulfilled)**  **Parameter 2:**  No documented human cases. **(Parameter 2 unfulfilled)**  **Parameter 3:**  No cases detected in cattle and livestock.(137) **(Parameter 3 unfulfilled)**  **Parameter 4:**  Cattle and livestock, both present in Nigeria.(138) **(Parameter 4 unfulfilled)**  **Parameter 5:**  Ecological suitability throughout northern Nigeria demonstrated through ecological niche modeling.(139) **(Parameter 5 fulfilled)** |
| *Bartonella* spp. | **Parameter 1:**  Human-to-human transmission of Bartonella species is rare. Although uncommon, transmission via blood transfusion has been reported for certain Bartonella species.(140) Most infections occur through arthropod vectors such as fleas, lice, and sandflies(141) or through scratches and bites. **(Parameter 1 partially fulfilled)**  **Parameter 2:**  No documented detection in humans in Nigeria. **(Parameter 2 unfulfilled)**  **Parameter 3**:  A 2014 study screened bats and bat flies captured in sites across Nigeria and molecularly detected *Bartonella* spp. in 51.4% and 41.7%, respectively.(142) **(Parameter 3a fulfilled)** |
| Bordetella pertussis (Pertussis, Whooping Cough) | **Parameter 1:**  Transmitted from human to human through respiratory aerosols and airborne droplets.(143) **(Parameter 1 fulfilled)** |
| *Brucella* spp. | **Parameter 1:**  Brucella species are primarily transmitted to humans through direct contact with infected animals, consumption of contaminated animal products, or by inhaling brucella.(144) While human-to-human transmission is rare, isolated cases via breastfeeding,(145) sexual contact,(146) and blood transfusions(147) have been described. **(Parameter 1 partially fulfilled)**  **Parameter 2:**  Known endemicity among humans and animals in Nigeria.(148) **(Parameter 2a fulfilled)** |
| *Burkholderia mallei* (glanders) | **Parameter 1:**  Typically associated with soliped mammals like horses, donkeys, or mules. Rare cases of human transmission primarily occur via direct contact with infected soliped bodily fluids, including through skin abrasions, inhalation of aerosols, or mucosal exposure.(149) **(Parameter 1 partially fulfilled)**  **Parameters 2-3:**  No modern day cases detected in Nigeria, or anywhere on the African continent.(150) **(Parameters 2-3 unfulfilled)**  **Parameter 4:**  There are horses(151) and donkeys(152) in Nigeria. **(Parameter 4 fulfilled)** |
| *Burkholderia pseudomallei* (melioidosis) | **Parameter 1:**  Transmission to humans typically occurs through percutaneous inoculation, inhalation of aerosolized bacteria, or ingestion of contaminated water or food.  Human-to-human transmission is rare, with only a few isolated cases reported where transmission occurred via sexual contact or nosocomial routes.(153) **(Parameter 1 partially fulfilled)**  **Parameters 2-3:**  No clear evidence of molecular or serological detection in Nigeria.(154) **(Parameters 2-3 unfulfilled)**  **Parameters 4-5:**  **(Parameters 4-5 not applicable)** |
| *Campylobacter jejuni* | **Parameter 1:**  Transmission to humans is typically via contaminated food, especially undercooked poultry, and sometimes through untreated water or unpasteurized milk.(155) **(Parameter 1 unfulfilled)**  **Parameters 2-3:**  Multiple studies report molecular detection in humans(156) and animals (157) in Nigeria. **(Parameters 2a-3a fulfilled)** |
| *Chlamydia psittaci* (Psittacosis) | **Parameter 1:**  Is typically associated with transmitted to humans via various birds species, including *Psittacidae* (parrots, parakeets, & cockatoos) and *Columbiformes* (pigeons).(158) However, human-to-human transmission through close personal contact and nosocomial routes has been documented.(159) **(Parameter 1 partially fulfilled)**  **Parameters 2-3:**  Primarily detected in Asia, Europe, Oceania, and the Americas. Reported cases in Africa are rare.(160) There have been no detections in Nigeria in animals or humans.(160,161) **(Parameters 2-3 unfulfilled)**  **Parameter 4:**  The true range of reservoirs is unknown; however, most cases are associated with domesticated avian species, other domesticated animals, or farm animals. It has also been found in wild birds, such as eagles and doves, and feral pigeons in urban areas are a natural reservoir of *C. psittaci.*(162) Both domesticated and farm animals are found in Nigeria. Various eagle, dove, and parrot species can be found in Nigeria.(163) **(Parameter 4 fulfilled)** |
| *Coxiella burnetii* | **Parameter 1:**  Human-to-human transmission is rare.(164) Most infections occur through inhalation of contaminated aerosols from infected animals or their products.(165) However, rare instances of transmission via blood transfusion(166) or perinatal(167) routes have been documented. **(Parameter 1 partially fulfilled)**  **Parameter 2:**  Serological detection among pastoralists in Sokoto State, Nigeria, revealed 62.57% and 2.98% seropositivity among the pastoralists and their cattle, respectively.(168) **(Parameter 2b fulfilled)** |
| *Clostridium botulinum* (Botulism) | **Parameter 1:**  Transmission occurs by the fecal-oral route.(169,170) **(Parameter 1 fulfilled)** |
| *Corynebacterium diphtheriae* (Diphtheria) | **Parameter 1:**  Transmitted by physical contact via respiratory secretions from a patient or a carrier.(171) **(Parameter 1 fulfilled)** |
| Diarrheagenic *E.coli* | **Parameter 1:**  Transmission to humans typically occurs through the fecal-oral route.(172) **(Parameter 1 fulfilled)** |
| *Francisella tularensis* (Tularemia) | **Parameter 1:**  Main sources of infection for humans include lagomorphs and small rodents, or through the skin via contact with an infected animal or arthropod bites(173) and by *Culex* and *Culiseta* mosquitoes.(174) **(Parameter 1 unfulfilled)**  **Parameters 2-3:**  No human or animal cases recorded anywhere in the southern hemisphere.(175) **(Parameters 2-3 unfulfilled)**  **Parameter 4:**  Its vectors and reservoirs infect multiple taxa. The geographic distribution of the animal species found in the wild to be infected with it does not include Africa,(175) with the exception of the following two species: *Mus musculus:* Found in Nigeria.(176) *Lepus* spp.: *Leptus victoriae* found in Nigeria.(177) **(Parameter 4 fulfilled)** |
| *Leptospira* spp | **Parameter 1:**  Human-to-human transmission of Leptospira species is rare.(178) Most infections result from direct contact with water, soil, or food contaminated with urine from infected animals.(179) **(Parameter 1 partially fulfilled)**  **Parameters 2-3:**  A 2024 study examined blood specimens from abattoir workers, rats, and cattle from Plateau state, Nigeria. *Leptospira* spp. was molecularly detected in 13.96 % (humans), 13.45 % (rats), and 6.35 % (cattle).(180) **(Parameters 2a and 3a fulfilled)** |
| *Listeria monocytogenes* | **Parameter 1:**  Transmission routes to humans can occur via blood transfusions, contaminated food(172), and maternal-neonatal routes.(181) **(Parameter 1 fulfilled)** |
| Mycobacterium tuberculosis (Tuberculosis) | **Parameter 1:**  Airborne human-to-human transmission.(182) **(Parameter 1 fulfilled)** |
| *Mycobacterium ulcerans* (Buruli Ulcers) | **Parameter 1:**  Unknown mode of transmission (183) and is not considered to be transmissible from human to human.(184) **(Parameter 1 unfulfilled)**  **Parameter 2:**  Documented human cases in Nigeria.(185) **(Parameter 2 fulfilled**) |
| *Neisseria meningitidis* (Meningococcus disease) | **Parameter 1:**  Human-to-human transmission via direct contact or through dispersion of respiratory droplets from a carrier(186) and through sexual transmission.(187) **(Parameter 1 fulfilled)** |
| *Orientia tsutsugamushi* | **Parameter 1:**  In the Asian-Pacific region it is primarily transmitted to humans via a chigger mite vector, *Leptotrombidium* sp*.*(188) **(Parameter 1 unfulfilled)**  **Parameters 2-3:**  Current known geographic distribution does not include the African continent. It is mainly transmitted in the “Tsutsugamushi Triangle,” which includes parts of multiple countries including Japan, Russia, Australia, Pakistan, Afghanistan, the Western Pacific islands, and China.(189) **(Parameters 2-3 unfulfilled)**  **Parameter 4:**  The known distribution of *Leptotrombidium sp.* does not include Nigeria or any country on the African continent.(190) **(Parameter 4 unfulfilled)**  **Parameter 5:**  The main vector species are *Leptotrombidium delicense, L. pallidum* and *L. scutellare.*(191)  *L. scutellare* are mainly found in mountainous areas at high altitudes (>3500 m).(192) No defined habitat requirements for *L. pallidum* were found. Maximum elevation in Nigeria is under 2,000 m.(193) The complete habitat requirements of *L. deliense* are unknown; however, they are commonly found on plains, flatlands, and in valleys at low altitudes. Their lifecycle can be completed in the temperature range of 13 ± 1–35 ± 1°C. There are plains, flatlands, and valleys in Nigeria,(177) and this temperature range can be found in Nigeria.(25) (**Parameter 5 fulfilled)** |
| Pan-Salmonella | **Parameter 1:**  While there have been rare cases of non–typhoidal *Salmonellae* (NTS) transmission between humans,(194) Salmonella transmission to humans is more commonly attributed to pets, farm animals, or water (in cases of Newport, Typhimurium, Saintpaul, and Dublin *Salmonella* serotypes).(195) **(Parameter 1 partially fulfilled)**  **Parameter 2:**  Typhoid fever remains a significant public health issue in low- and middle-income countries and has a known endemicity in Nigeria.(196) **(Parameter 2a fulfilled)** |
| *Rickettsia sp.* | **Parameter 1:**  Transmission of rickettsiae to humans is primarily via the bite of infected arthropods such as ticks, fleas, mites, and lice. Infection is also possible through inhalation of airborne bacteria (in *Rickettsia prowazekii)*, or when bacteria present in arthropod feces enter the body through bite wounds, the eyes, or mucous membranes (in *Rickettsia typhi)*.(197) **(Parameter 1 unfulfilled)**  **Parameter 2:**  No documented human cases of any Rickettsia spp. in Nigeria.(198) **(Parameter 2 unfulfilled)**  **Parameter 3:**  Molecular detection in dogs and ticks (199) and ticks from the vegetation and livestock in Nigeria.(200) **(Parameter 3a fulfilled)** |
| *Shigella sp.* (Shigellosis) | **Parameter 1:**  Primarily transmitted via fecal-oral route with *Musca* domestica and through direct contact or consumption of food and water.(201,202) **(Parameter 1 fulfilled)** |
| *Streptococcus pneumoniae* | **Parameter 1:**  Transmission between humans typically occurs through respiratory routes.(203) **(Parameter 1 fulfilled)** |
| *Vibrio cholerae* (Cholera) | **Parameter 1:**  Transmitted fecal-orally either directly from human to human or from consumption of contaminated food/drink.(204,205) **(Parameter 1 fulfilled)** |
| *Yersinia enterocolitica* | **Parameter 1:**  Transmission to humans primarily occurs via food contaminated by the *Y. pestis, Y. pseudotuberculosis,* or *Y. enterocolitica* genera of *Yersinia enterocolitica.* Studies have suggested that human-to-human transmission through blood transfusion is possible.(206) **(Parameter 1 partially fulfilled)**  **Parameters 2-3:**  Molecular evidence in humans in Nigeria.(207) Has also molecularly detected in pigs, cattle, and sheep(207–209) in Nigeria. **(Parameters 2-3 fulfilled)** *Note:* *Yersinia enterocolitica* detected via culture. |
| *Yersinia pestis* (Plague) | **Parameter 1:**  Carried by rodents and transmitted within them through a flea vector (*Xenopsylla cheopis*).(210,211) **(Parameter 1 unfulfilled)**  **Parameters 2-3:**  No human or animal cases detected.(212) **(Parameters 2-3 unfulfilled)**  **Parameter 4:**  Found in small numbers 30+ years ago(213) but not detected since.(214) **(Parameter 4 unfulfilled)**  **Parameter 5:**  Potential distribution of plague in Nigeria based ecological niche modeling of environmental covariates including potential evapotranspiration, normalized difference in vegetation index of various years, and minimum and maximum temperatures of the coldest and hottest months, respectively.(215) **(Parameter 5 fulfilled)** |
| **Nematode Pathogens** | |
| Dracunculus medinensis (Guinea Worm) | **Parameter 1:**  Transmitted to humans by drinking water contaminated with crustaceans carrying the nematode.(216) **(Parameter 1 unfulfilled)**  **Parameter 2:**  Previous detection in humans.(217,218) **(Parameter 2a fulfilled)** |
| Wuchereria bancrofti, Brugia malayi, and Brugia timori (Lymphatic Filariasis) | **Parameter 1:**  Transmitted to humans via infected mosquito bite from the following species: Anopheles, Aedes, Culex, and Mansonia.(219) **(Parameter 1 unfulfilled)**  **Parameter 2:**  Human cases detected throughout Nigeria.(220,221) **(Parameter 2a fulfilled)** *Note: Cases detected through antigen tests.* |
| **Protozoan Pathogens** | |
| *Balamuthia mandrillaris* | **Parameter 1:**  The most common route of infection is through exposure to contaminated soil, via interaction with dirt or gardening, and inhalation of cysts carried by the wind, are likely sources of infection.(222) Human-to-human transmission can occur through organ transplantation and through inhalation, but this is not the most common route of infection.(223) **(Parameter 1 partially fulfilled)**  **Parameters 2-3:**  No human or animal cases reported in Nigeria.(224) **(Parameters 2-3 unfulfilled)**  **P**a**rameters 4-5:**  Limited knowledge about the ecological distribution of these amoebae, although they have been isolated from the soil.(224,225) Are considered to be free-living amoebae.(226) **(Parameters 4-5 not applicable)** |
| *Cryptosporidium parvum* | **Parameter 1:**  Transmission typically occurs through the oral-fecal route through consumption of contaminated food or water.(227) **(Parameter 1 fulfilled)** |
| *Cyclospora cayatanensis* | **Parameter 1:**  Transmission in humans is typically associated with fecal contact, especially via consumption of contaminated food and water.(228) **(Parameter 1 fulfilled)** |
| *Giardia lamblia* | **Parameter 1:**  Transmission to humans typically occurs through consumption of contaminated water or food. Human-to-human transmission also occurs via sexual and direct fecal contact. (229) **(Parameter 1 fulfilled)** |
| *Leishmania* spp. | **Parameter 1:**  Transmission to humans usually occurs via bites from infected female sandflies (genera *Phlebotomus* and *Lutzomyia*).(230) Rare routes of transmission between humans include congenital transmission, blood transfusion, organ transplantation, and, in rare cases, sexual transmission.(231) **(Parameter 1 partially fulfilled)**  **Parameter 2:**  Has a known endemicity in Nigeria.(232) **(Parameter 2a fulfilled)** |
| Microsporidia | **Parameter 1:**  Transmission to humans occurs via transplantation, respiratory, and sexual contact.(233) **(Parameter 1 fulfilled)** |
| *Plasmodium* sp. | **Parameter 1:**  Transmission to humans primarily occurs through bites from infected *Anopheles* sp. mosquitoes.(234) However, transmission between humans can occur through blood transfusions, congenital transmission (mother to fetus), and organ transplants.(235) The major species causing malaria in humans are *P. falciparum*, *P. vivax*, *P. ovale*, *P. malariae*, and *P. knowlesi*.(235) **(Parameter 1 partially fulfilled)**  **Parameter 2:**  *Plasmodium* sp. has a known endemicity throughout parts of Africa, with Nigeria reporting the highest malaria burden globally.(236) (**Parameter 2a fulfilled)** |
| *Trypanosoma brucei* | **Parameter 1:**  Transmission of *Trypanosoma brucei* to humans typically occurs via bites from infected tsetse flies (genus *Glossina*).(237) In humans, the disease is caused by the *T. b. gambiense* and *T. b. rhodesiense* subspecies.(238) Human-to-human transmission is less common, often through congenital, blood transfusions, and organ transplantation routes.(239) **(Parameter 1 partially fulfilled)**  **Parameter 2:**  Is targeted for elimination in Nigeria; however, there remain some cases and silent transmission.(240) **(Parameter 2a fulfilled)** |
| *Naegleria fowleri* | **Parameter 1:**  Transmission primarily occurs through intranasal exposure to contaminated water sources. (241) **(Parameter 1 fulfilled)** |
| *Toxoplasma gondii* | **Parameter 1:**  *Toxoplasma gondii* is transmitted to humans primarily through ingestion of meat, soil, water, or food containing oocysts.(242) **(Parameter 1 unfulfilled)**  **Parameter 2:**  A 2020 meta-analysis reported on detection in 17 Nigerian states (only molecular detection in Oyo state) and moderate prevalence across the country.(243) **(Parameter 2b fulfilled)** |

­­­

**References**

1. D’Souza DH, Sair A, Williams K, Papafragkou E, Jean J, Moore C, et al. Persistence of caliciviruses on environmental surfaces and their transfer to food. International Journal of Food Microbiology. 2006 Apr;108(1):84–91.

2. Rotavirus and Noro- and Caliciviruses. In: Textbook of Clinical Pediatrics [Internet]. Berlin, Heidelberg: Springer Berlin Heidelberg; 2012 [cited 2025 Jul 25]. p. 1249–57. Available from: http://link.springer.com/10.1007/978-3-642-02202-9_120

3. Radoshitzky SR, Kuhn JH, Jahrling PB, Bavari S. Hemorrhagic fever-causing mammarenaviruses. Medical Aspects of Biological Warfare; Bozue, J, Cote, CK, Glass, PJ, Eds. 2018;517–45.

4. Salvato MS. The Arenaviridae. Illustrated. Springer Science & Business Media; 2012. 401 p. (The Viruses).

5. Delgado S, Erickson BR, Agudo R, Blair PJ, Vallejo E, Albariño CG, et al. Chapare Virus, a Newly Discovered Arenavirus Isolated from a Fatal Hemorrhagic Fever Case in Bolivia. PLOS Pathogens. 2008 Apr 18;4(4):e1000047.

6. Escalera-Antezana JP, Rodriguez-Villena OJ, Arancibia-Alba AW, Alvarado-Arnez LE, Bonilla-Aldana DK, Rodríguez-Morales AJ. Clinical features of fatal cases of Chapare virus hemorrhagic fever originating from rural La Paz, Bolivia, 2019: A cluster analysis. Travel medicine and infectious disease. 2020;36:101589.

7. Cossaboom C, Ramirez AM, Romero C, Morales-Betoulle M, De la Vega G, Gutiérrez J, et al. Re-emergence of Chapare hemorrhagic fever in Bolivia, 2019. International Journal of Infectious Diseases. 2020;101:244–5.

8. Sarute N, Ross SR. New World arenavirus biology. Annual review of virology. 2017;4:141–58.

9. World Health Organization, UNICEF. Global vector control response 2017-2030. 2017;

10. Abdullahi IN, Akande AO, Muhammed Y, Rogo LD, Oderinde BS. Prevalence Pattern of Chikungunya Virus Infection in Nigeria: A Four Decade Systematic Review and Meta-analysis. Pathog Glob Health. 2020 May;114(3):111–6.

11. Omatola CA, Onoja BA, Fassan PK, Osaruyi SA, Iyeh M, Samuel MA, et al. Seroprevalence of chikungunya virus infection in five hospitals within Anyigba, Kogi State of Nigeria. Braz J Infect Dis. 2020 Feb;24(1):1–6.

12. Bente DA, Forrester NL, Watts DM, McAuley AJ, Whitehouse CA, Bray M. Crimean-Congo hemorrhagic fever: history, epidemiology, pathogenesis, clinical syndrome and genetic diversity. Antiviral research. 2013;100(1):159–89.

13. Bukbuk DN, Dowall SD, Lewandowski K, Bosworth A, Baba SS, Varghese A, et al. Serological and virological evidence of Crimean-Congo haemorrhagic fever virus circulation in the human population of Borno State, northeastern Nigeria. PLoS Neglected Tropical Diseases. 2016;10(12):e0005126.

14. Woodall J, Williams M, Simpson D. Congo virus: a hitherto undescribed virus occurring in Africa. Part 2. Identification studies. East African medical journal. 1967;44(2):93–8.

15. Oluwayelu D, Afrough B, Adebiyi A, Varghese A, Eun-Sil P, Fukushi S, et al. Prevalence of antibodies to Crimean-Congo hemorrhagic fever virus in ruminants, Nigeria, 2015. Emerging infectious diseases. 2020;26(4):744.

16. Otu A, Ebenso B, Etokidem A, Chukwuekezie O. Dengue fever–an update review and implications for Nigeria, and similar countries. African Health Sciences. 2019;19(2):2000–7.

17. Fagbami AH, Onoja AB. Dengue haemorrhagic fever: An emerging disease in Nigeria, West Africa. Journal of infection and public health. 2018;11(6):757–62.

18. Ayukekbong JA. Dengue virus in Nigeria: Current status and future perspective. Hosts and Viruses. 2014;1(4):106.

19. Armstrong PM, Andreadis TG. Eastern Equine Encephalitis Virus — Old Enemy, New Threat. N Engl J Med. 2013 May 2;368(18):1670–3.

20. Kumar B, Manuja A, Gulati BR, Virmani N, Tripathi BN. Zoonotic Viral Diseases of Equines and Their Impact on Human and Animal Health. Open Virol J. 2018;12:80–98.

21. Martínez-Barciela Y, González AP, Rial DG, González JG. First records of five species of mosquitoes (Diptera: Culicidae) in Galicia, including the first evidence of the genus Coquillettidia in northwestern Spain. Journal of Vector Ecology [Internet]. 2021 Jun 14 [cited 2025 Jul 22];46(1). Available from: https://bioone.org/journals/journal-of-vector-ecology/volume-46/issue-1/1081-1710-46.1.96/First-records-of-five-species-of-mosquitoes-Diptera--Culicidae/10.52707/1081-1710-46.1.96.full

22. Mahmood F, Crans WJ. Ovarian development and parity determination in Culiseta melanura (Diptera: Culicidae). J Med Entomol. 1998 Nov;35(6):980–8.

23. Igu NI. Species Diversity and Structure of an Intact Freshwater Swamp Forest in the Niger Delta. OJF. 2017;07(02):242–54.

24. Sallam MF, Whitehead S, Barve N, Bauer A, Guralnick R, Allen J, et al. Co-occurrence probabilities between mosquito vectors of West Nile and Eastern equine encephalitis viruses using Markov Random Fields (MRFcov). Parasites Vectors. 2023 Jan 10;16(1):10.

25. Williams E, Funk C, Peterson P, Tuholske C. High resolution climate change observations and projections for the evaluation of heat-related extremes. Sci Data. 2024 Mar 1;11(1):261.

26. Judson S, Prescott J, Munster V. Understanding Ebola virus transmission. Viruses. 2015;7(2):511–21.

27. Schindell BG, Webb AL, Kindrachuk J. Persistence and sexual transmission of filoviruses. Viruses. 2018;10(12):683.

28. Silva-Ramos CR, Montoya-Ruíz C, Faccini-Martínez ÁA, Rodas JD. An updated review and current challenges of Guanarito virus infection, Venezuelan hemorrhagic fever. Archives of Virology. 2022;167(9):1727–38.

29. Manzione N de, Salas RA, Paredes H, Godoy O, Rojas L, Araoz F, et al. Venezuelan hemorrhagic fever: clinical and epidemiological studies of 165 cases. Clinical infectious diseases. 1998;26(2):308–13.

30. Fulhorst CF, Bowen MD, Salas RA, Duno G, Utrera A, Ksiazek TG, et al. Natural rodent host associations of Guanarito and pirital viruses (Family Arenaviridae) in central Venezuela. The American journal of tropical medicine and hygiene. 1999;61(2):325–30.

31. Tsendbazar NE, Tarko A, Linlin L, Herold M, Lesiv M, Fritz S, et al. Copernicus Global Land Service: Land Cover 100m: Version 3 Globe 2015-2019: Validation Report [Internet]. 2020 [cited 2024 Jun 17]. Available from: file:///C:/Users/cquiner/Downloads/CGLOPS1_VR_LC100m-V3.0_I1.00_revised_202103.pdf

32. Zanaga D, Van De Kerchove R, Daems D, De Keersmaecker W, Brockmann C, Kirches G, et al. ESA WorldCover 10 m 2021 v200. 2022;

33. Voss R. An introduction to the neotropical muroid rodent genus Zygodontomys. In New York: [New York] : American Museum of Natural History; 1991 [cited 2024 Mar 21]. p. 113. (Bulletin of the American Museum of Natural History). Available from: https://digitallibrary.amnh.org/bitstreams/1b92b2ab-a5b6-4337-87fe-c98507a32d35/download

34. Jarvis A, Reuter HI, Nelson A, Guevara E. Hole-filled SRTM for the globe Version 4. available from the CGIAR-CSI SRTM 90m Database (http://srtm csi cgiar org). 2008;15(25–54):5.

35. Adedoyin OS, Aluko SO, Olabode MA, Ajibola AA, Ajagun Ebunoluwa Oghale, Ajakaye Oludare Clement. Global Forest Resources assessment 2015. Country Report. Nigeria [Internet]. Food and Agriculture Organization; 2014 [cited 2023 Sep 21]. Available from: https://www.fao.org/3/az293e/az293e.pdf

36. Gallant AL, Klaver RW, Casper GS, Lannoo MJ. Global Rates of Habitat Loss and Implications for Amphibian Conservation. Copeia. 2007 Dec 28;2007(4):967–79.

37. Toledo J, Haby MM, Reveiz L, Sosa Leon L, Angerami R, Aldighieri S. Evidence for human-to-human transmission of hantavirus: a systematic review. The Journal of infectious diseases. 2022;226(8):1362–71.

38. Klempa B, Koivogui L, Sylla O, Koulemou K, Auste B, Krüger DH, et al. Serological evidence of human hantavirus infections in Guinea, West Africa. The Journal of infectious diseases. 2010;201(7):1031–4.

39. Klempa B, Fichet-Calvet E, Lecompte E, Auste B, Aniskin V, Meisel H, et al. Hantavirus in African wood mouse, Guinea. Emerging infectious diseases. 2006;12(5):838.

40. Gu SH, Nicolas V, Lalis A, Sathirapongsasuti N, Yanagihara R. Complete genome sequence and molecular phylogeny of a newfound hantavirus harbored by the Doucet’s musk shrew (Crocidura douceti) in Guinea. Infection, Genetics and Evolution. 2013;20:118–23.

41. Kang HJ, Kadjo B, Dubey S, Jacquet F, Yanagihara R. Molecular evolution of Azagny virus, a newfound hantavirus harbored by the West African pygmy shrew (Crocidura obscurior) in Cote d’Ivoire. Virology Journal. 2011;8:1–7.

42. Igbokwe J, Nicolas V, Oyeyiola A, Obadare A, Adesina AS, Awodiran MO, et al. Molecular taxonomy of Crocidura species (Eulipotyphla: Soricidae) in a key biogeographical region for African shrews, Nigeria. Comptes rendus biologies. 2019;342(3–4):108–17.

43. Nainan OV, Xia G, Vaughan G, Margolis HS. Diagnosis of Hepatitis A Virus Infection: a Molecular Approach. Clin Microbiol Rev. 2006 Jan;19(1):63–79.

44. Li P, Liu J, Li Y, Su J, Ma Z, Bramer WM, et al. The global epidemiology of hepatitis E virus infection: A systematic review and meta-analysis. Liver Int. 2020 Jul;40(7):1516–28.

45. Meng XJ. Zoonotic and Foodborne Transmission of Hepatitis E Virus. Semin Liver Dis. 2013 Apr 5;33(01):041–9.

46. Kumar S, Subhadra S, Singh B, Panda BK. Hepatitis E virus: the current scenario. International Journal of Infectious Diseases. 2013 Apr;17(4):e228–33.

47. Meng XJ. From barnyard to food table: The omnipresence of hepatitis E virus and risk for zoonotic infection and food safety. Virus Research. 2011 Oct;161(1):23–30.

48. Srivastava KS, Jeswani V, Pal N, Bohra B, Vishwakarma V, Bapat AA, et al. Japanese Encephalitis Virus: An Update on the Potential Antivirals and Vaccines. Vaccines. 2023 Mar 27;11(4):742.

49. Kuwata R, Torii S, Shimoda H, Supriyono S, Phichitraslip T, Prasertsincharoen N, et al. Distribution of Japanese Encephalitis Virus, Japan and Southeast Asia, 2016-2018. Emerg Infect Dis. 2020 Jan;26(1):125–8.

50. Adeleke R, Olanipekun T, Abiola J, Aluko A, Sule W, Oluwayelu D. Serological Investigation of Japanese Encephalitis Virus Infection in Commercially Reared Pigs, Southwestern Nigeria. Vet Ital. 2023 Dec 31;59(4).

51. MacLachlan NJ, Dubovi EJ, editors. Chapter 23 - Arenaviridae. In: Fenner’s Veterinary Virology (Fifth Edition) [Internet]. Boston: Academic Press; 2017 [cited 2025 Aug 1]. p. 425–34. Available from: https://www.sciencedirect.com/science/article/pii/B9780128009468000234

52. Flores-Pérez N, Kulkarni P, Uhart M, Pandit PS. Climate Change Impact on Human-Rodent Interfaces: Modeling Junin Virus Reservoir Shifts. EcoHealth [Internet]. 2025 Jun 27 [cited 2025 Jul 15]; Available from: https://link.springer.com/10.1007/s10393-025-01723-z

53. González Ittig RE, Gardenal CN. Recent range expansion and low levels of contemporary gene flow in Calomys musculinus: its relationship with the emergence and spread of Argentine haemorrhagic fever. Heredity (Edinb). 2004 Dec;93(6):535–41.

54. IUCN. Calomys laucha: Christoff, A., Weksler, M., Vieira, E, D’Elia, G., Jayat., J.P. & Pardinas., U.: The IUCN Red List of Threatened Species 2008: e.T3613A9983560 [Internet]. 2008 [cited 2025 Aug 1]. Available from: https://www.iucnredlist.org/species/3613/9983560

55. Abba AM, Varela D, Cirignoli S, Pereira JA, Bolkovic ML, Peker S, et al. Categorización de los mamíferos de Argentina 2019: Resumen y análisis de las amenazas. Mastozoología neotropical. 2022;29(1):657–657.

56. Mills JN, Ellis BA, McKee KT, Calderon GE, Maiztegui JI, Nelson GO, et al. A Longitudinal Study of Junin Virus Activity in the Rodent Reservoir of Agrentine Hemorrhagic Fever. The American Journal of Tropical Medicine and Hygiene. 1992 Dec;47(6):749–63.

57. Leandro Parente, Lindsey Sloat, Vinicius Mesquita, Davide Consoli, Radost Stanimirova, Tomislav Hengl, et al. Global Pasture Watch - Annual grassland class and extent maps at 30-m spatial resolution (2000—2022) [Internet]. Zenodo; 2024 [cited 2025 Aug 1]. Available from: https://zenodo.org/doi/10.5281/zenodo.13890401

58. Parente L, Sloat L, Mesquita V, Consoli D, Stanimirova R, Hengl T, et al. Annual 30-m maps of global grassland class and extent (2000–2022) based on spatiotemporal Machine Learning. Sci Data. 2024 Dec 11;11(1):1303.

59. Leisnham PT, Juliano SA. Impacts of Climate, Land Use, and Biological Invasion on the Ecology of Immature Aedes Mosquitoes: Implications for La Crosse Emergence. EcoHealth. 2012 Jun;9(2):217–28.

60. Khan UM, Gudlavalleti A. La Crosse Encephalitis. In: StatPearls [Internet]. Treasure Island (FL): StatPearls Publishing; 2025 [cited 2025 Jul 25]. Available from: http://www.ncbi.nlm.nih.gov/books/NBK562248/

61. Harding S, Greig J, Mascarenhas M, Young I, Waddell LA. La Crosse virus: a scoping review of the global evidence. Epidemiol Infect. 2018 Dec 5;147:e66.

62. Babalola AS, Adeogun AO, Thabet HS, TagEldin RA, Oyeniyi T, Adekunle O, et al. Geospatial modeling of geographical spread of Aedes species, in relation to climatic and topographical factors in Lagos State, Nigeria. PLoS Negl Trop Dis. 2025 Feb;19(2):e0012860.

63. Saluzzo J, Dodet B. Emergence and control of rodent-borne viral diseases. 1st ed. Elsevier; 1999. 289 p.

64. Ogbu O, Ajuluchukwu E, Uneke C. Lassa fever in West African sub-region: an overview. Journal of vector borne diseases. 2007;44(1):1.

65. Fichet-Calvet E, Rogers DJ. Risk maps of Lassa fever in West Africa. PLoS neglected tropical diseases. 2009;3(3):e388.

66. McCormick J, Fisher-Hoch S. Areanaviruses I: Lassa fever. Pringer; 2002. (Current Topics in Microbiology and Immunology; vol. 262).

67. Briese T, Paweska JT, McMullan LK, Hutchison SK, Street C, Palacios G, et al. Genetic detection and characterization of Lujo virus, a new hemorrhagic fever–associated arenavirus from Southern Africa. PLoS pathogens. 2009;5(5):e1000455.

68. Sewlall NH, Paweska JT. Lujo virus: current concepts. Virus Adaptation and Treatment. 2017;41–7.

69. Paweska JT, Sewlall NH, Ksiazek TG, Blumberg LH, Hale MJ, Lipkin WI, et al. Nosocomial outbreak of novel arenavirus infection, southern Africa. Emerging infectious diseases. 2009;15(10):1598.

70. Johnson KM, Kuns ML, Mackenzie RB, Webb PA, Yunker CE. Isolation of Machupo virus from wild rodent Calomys callosus. American Journal of Tropical Medicine and Hygiene. 1966;15(1):103–6.

71. Mackenzie RB, Beye HK, Valverde CL, Garrón H. Epidemic Hemorrhagic Fever in Bolivia. I. A Preliminary Report of the Epide-miologic and Clinical Findings in a New Epidemic Area in South America. American Journal of Tropical Medicine and Hygiene. 1964;13(4):620–5.

72. Aguilar PV, Camargo W, Vargas J, Guevara C, Roca Y, Vidal Felices V, et al. Reemergence of Bolivian hemorrhagic fever, 2007–2008. Emerging infectious diseases. 2009;15(9):1526.

73. Dunnum, J, Vargas, J, Bernal, N, D’elia, G, Pardinas, U, Teta, P. Calomys callosus. The IUCN Red List of Threatened Species. [Internet]. 2016 [cited 2023 Sep 21]. Available from: https://dx.doi.org/10.2305/IUCN.UK.2016-3.RLTS.T3611A22334720.en.

74. Vainrub B, Salas R. Latin American hemorrhagic fever. Infectious Disease Clinics of North America. 1994;8(1):47–59.

75. Salazar-Bravo J, Dragoo JW, Bowen MD, Peters CJ, Ksiazek TG, Yates TL. Natural nidality in Bolivian hemorrhagic fever and the systematics of the reservoir species. Infection, Genetics and Evolution. 2002;1(3):191–9.

76. Espinosa M, Lasserre A, Piantanida M, Vitullo A. Cytogenetics of vesper mice, Calomys (Sigmodontinae): a new karyotype from the Puna region and its implication for chromosomal phylogeny. Cellular and Molecular Life Sciences CMLS. 1997;53:583–6.

77. Adjemian J, Farnon EC, Tschioko F, Wamala JF, Byaruhanga E, Bwire GS, et al. Outbreak of Marburg hemorrhagic fever among miners in Kamwenge and Ibanda Districts, Uganda, 2007. The Journal of infectious diseases. 2011;204(suppl_3):S796–9.

78. Towner JS, Khristova ML, Sealy TK, Vincent MJ, Erickson BR, Bawiec DA, et al. Marburgvirus genomics and association with a large hemorrhagic fever outbreak in Angola. Journal of virology. 2006;80(13):6497–516.

79. Bausch DG, Nichol ST, Muyembe-Tamfum JJ, Borchert M, Rollin PE, Sleurs H, et al. Marburg hemorrhagic fever associated with multiple genetic lineages of virus. New England Journal of Medicine. 2006;355(9):909–19.

80. World Health Organization. West Africa’s first-ever case of Marburg virus disease confirmed in Guinea. 2021 Aug 9 [cited 2023 Sep 12]; Available from: https://www.afro.who.int/news/west-africas-first-ever-case-marburg-virus-disease-confirmed-guinea

81. Kortepeter MG, Dierberg K, Shenoy ES, Cieslak TJ. Marburg virus disease: A summary for clinicians. Int J Infect Dis. 2020 Oct;99:233–42.

82. Kwiecinski GG, Griffiths TA. Rousettus egyptiacus. Mammalian species. 1999;(611):1–9.

83. Pigott DM, Golding N, Mylne A, Huang Z, Weiss DJ, Brady OJ, et al. Mapping the zoonotic niche of Marburg virus disease in Africa. Transactions of the Royal Society of Tropical Medicine and Hygiene. 2015;109(6):366–78.

84. Hutson CL, Olson VA, Carroll DS, Abel JA, Hughes CM, Braden ZH, et al. A prairie dog animal model of systemic orthopoxvirus disease using West African and Congo Basin strains of monkeypox virus. J Gen Virol. 2009 Feb;90(Pt 2):323–33.

85. Jezek Z, Grab B, Szczeniowski MV, Paluku KM, Mutombo M. Human monkeypox: secondary attack rates. Bull World Health Organ. 1988;66(4):465–70.

86. Madhukalya R, Yadav U, Parray HA, Raj N, Lupitha SS, Kumar V, et al. Nipah virus: pathogenesis, genome, diagnosis, and treatment. Appl Microbiol Biotechnol. 2025 Jul 1;109(1):158.

87. Ganguly A, Mahapatra S, Ray S, Chattopadhyay S, Islam MdJ, Garai S, et al. The rising threat of Nipah virus: a highly contagious and deadly zoonotic pathogen. Virol J [Internet]. 2025 May 10 [cited 2025 Jul 22];22(1). Available from: https://virologyj.biomedcentral.com/articles/10.1186/s12985-025-02728-4

88. Adamu AM, McNabb L, Adikwu AA, Jibril YJ, Idoko SI, Turaki AU, et al. Henipavirus sero-surveillance in horses and pigs from Northern Nigeria. Front Virol. 2022 Aug 2;2:929715.

89. IUCN. Pteropus giganteus: Tsang, S.M.: The IUCN Red List of Threatened Species 2020: e.T18725A194134899 [Internet]. 2019 [cited 2025 Jul 29]. Available from: https://www.iucnredlist.org/species/18725/194134899

90. Madala MF, Guna C, Pradeepan A, Chalil AK. Roost tree characteristics of Pteropus medius (Chiroptera: Pteropodidae) in the midland laterite hillocks of northern Kerala, India. Journal of Asia-Pacific Biodiversity. 2022 Dec;15(4):465–72.

91. Rezza G, Chen R, Weaver SC. O’nyong-nyong fever: a neglected mosquito-borne viral disease. Pathogens and Global Health. 2017 Aug 18;111(6):271–5.

92. Mutsaers M, Engdahl CS, Wilkman L, Ahlm C, Evander M, Lwande OW. Vector competence of Anopheles stephensi for O’nyong-nyong virus: a risk for global virus spread. Parasit Vectors. 2023 Apr 17;16(1):133.

93. Jagtap S, Altinli M, Badusche M, Chevalier M, Becker N, Leggewie M, et al. Invasive Aedes albopictus is a competent vector for O’nyong Nyong virus. One Health. 2025 Jun;20:101062.

94. Okorie PN, McKenzie FE, Ademowo OG, Bockarie M, Kelly-Hope L. Nigeria Anopheles vector database: an overview of 100 years’ research. PLoS One. 2011;6(12):e28347.

95. Diaz JH. The Disease Ecology, Epidemiology, Clinical Manifestations, Management, Prevention, and Control of Increasing Human Infections with Animal Orthopoxviruses. Wilderness & Environmental Medicine. 2021 Dec;32(4):528–36.

96. Nathanson N, Kew OM. From emergence to eradication: the epidemiology of poliomyelitis deconstructed. Am J Epidemiol. 2010 Dec 1;172(11):1213–29.

97. Nnadi C, Damisa E, Esapa L, Braka F, Waziri N, Siddique A, et al. Continued Endemic Wild Poliovirus Transmission in Security-Compromised Areas - Nigeria, 2016. MMWR Morb Mortal Wkly Rep. 2017 Feb 24;66(7):190–3.

98. As Nigeria Celebrates 2 Years Of Polio Free: Emeka Offor Urges FG To Rema. This Day [Internet]. 2022 Oct 25 [cited 2023 Sep 21]; Available from: https://www.thisdaylive.com/index.php/2022/10/25/as-nigeria-celebrates-2-years-of-polio-free-emeka-offor-urges-fg-to-remain-vigilant

99. Rupprecht CE, Hanlon CA, Hemachudha T. Rabies re-examined. The Lancet infectious diseases. 2002;2(6):327–43.

100. Tang X, Luo M, Zhang S, Fooks AR, Hu R, Tu C. Pivotal role of dogs in rabies transmission, China. Emerging infectious diseases. 2005;11(12):1970.

101. Wertheim HFL, Nguyen TQ, Nguyen KAT, de Jong MD, Taylor WRJ, Le TV, et al. Furious rabies after an atypical exposure. PLoS medicine. 2009;6(3):e1000044.

102. Audu SW, Mshelbwala PP, Jahun BM, Bouaddi K, Weese JS. Two fatal cases of rabies in humans who did not receive rabies postexposure prophylaxis in Nigeria. Clinical case reports. 2019;7(4):749.

103. Mshelbwala PP, Weese JS, Sanni-Adeniyi OA, Chakma S, Okeme SS, Mamun AA, et al. Rabies epidemiology, prevention and control in Nigeria: Scoping progress towards elimination. PLoS Neglected Tropical Diseases. 2021;15(8):e0009617.

104. Monath TP. The Arboviruses:: Epidemiology and Ecology. Vol. 1. CRC Press; 2020.

105. Turell M, Bailey C, Beaman J. Vector competence of a Houston, Texas strain of Aedes albopictus for Rift Valley fever virus. Journal of the American Mosquito Control Association. 1988;4(1):94–6.

106. Turell MJ, Linthicum KJ, Patrican LA, Davies FG, Kairo A, Bailey CL. Vector competence of selected African mosquito (Diptera: Culicidae) species for Rift Valley fever virus. Journal of medical entomology. 2008;45(1):102–8.

107. Idris I, Adesola RO. The Existence of Rift Valley Fever Virus in Nigeria: Past, Present, And Future. Intersect: The Stanford Journal of Science, Technology, and Society. 2022;16(1).

108. Oragwa AO, Oragwa FC, Oluwayelu DO. Serologic evidence of silent Rift Valley fever virus infection among occupationally exposed persons in northern Nigeria. The Journal of Infection in Developing Countries. 2022;16(05):881–7.

109. Atuman YJ, Kudi CA, Abdu PA, Okubanjo OO, Wungak Y, Ularamu HG, et al. Serological evidence of antibodies to Rift Valley fever virus in wild and domestic animals in Bauchi State, Nigeria. Veterinary Medicine International. 2022;2022.

110. Adamu A, Enem S, Ngbede E, Owolodun O, Dzikwi A, Ajagbe O, et al. Serosurvey on sheep unravel circulation of Rift Valley fever virus in Nigeria. EcoHealth. 2020;17:393–7.

111. De Santis M, Cavaliere AF, Straface G, Caruso A. Rubella infection in pregnancy. Reproductive Toxicology. 2006;21(4):390–8.

112. De Vries RD, Duprex WP, De Swart RL. Morbillivirus infections: an introduction. Viruses. 2015;7(2):699–706.

113. Jean Baptiste AE, Masresha B, Wagai J, Luce R, Oteri J, Dieng B, et al. Trends in measles incidence and measles vaccination coverage in Nigeria, 2008–2018. Vaccine. 2021 Nov;39:C89–95.

114. World Health Organization. Transmission of SARS-CoV-2: implications for infection prevention precautions. [Internet]. 2020. Available from: www.who.int/news-room/commentaries/detail/transmission-of-sars-cov-2-implications-for-infection-prevention-precautions.

115. Ridenour CL, Cocking J, Poidmore S, Erickson D, Brock B, Valentine M, et al. St. Louis Encephalitis Virus in the Southwestern United States: A Phylogeographic Case for a Multi-Variant Introduction Event. Front Genet [Internet]. 2021 Jun 8 [cited 2025 Jul 25];12. Available from: https://www.frontiersin.org/articles/10.3389/fgene.2021.667895/full

116. Diaz A, Coffey LL, Burkett-Cadena N, Day JF. Reemergence of St. Louis Encephalitis Virus in the Americas. Emerg Infect Dis [Internet]. 2018 Dec [cited 2025 Jul 29];24(12). Available from: http://wwwnc.cdc.gov/eid/article/24/12/18-0372_article.htm

117. Mohammed BR, Yayo AM, Ajanusi OJ, Lawal IA. Relative abundance and molecular identification of Culex pipiens complex (Diptera: Culicidae), in Kura Local Government Area, North-western Nigeria. Parasite Epidemiol Control. 2021 Aug;14:e00213.

118. Omotayo AI, Dogara MM, Sufi D, Shuaibu T, Balogun J, Dawaki S, et al. High pyrethroid-resistance intensity in Culex quinquefasciatus (Say) (Diptera: Culicidae) populations from Jigawa, North-West, Nigeria. Lenhart A, editor. PLoS Negl Trop Dis. 2022 Jun 21;16(6):e0010525.

119. Weaver SC, Ferro C, Barrera R, Boshell J, Navarro JC. Venezuelan equine encephalitis. Annu Rev Entomol. 2004;49:141–74.

120. Crosby B, Crespo ME. Venezuelan Equine Encephalitis. In: StatPearls [Internet]. Treasure Island (FL): StatPearls Publishing; 2025 [cited 2025 Jul 25]. Available from: http://www.ncbi.nlm.nih.gov/books/NBK559332/

121. Nava MR, Debboun M. Chapter 2 - Mosquito Species of Texas. In: Debboun M, Nava MR, Rueda LM, editors. Mosquitoes, Communities, and Public Health in Texas [Internet]. Academic Press; 2020. p. 9–167. Available from: https://www.sciencedirect.com/science/article/pii/B978012814545600002X

122. UN Environment Programme-World Conservation Monitoring Centre (UNEP-WCMC) (2025). Ocean+ Habitats [Internet]. 2014 [cited 2025 Aug 1]. Available from: habitats.oceanplus.org

123. Ebenezer A, Noutcha AEM, Agi PI, Okiwelu SN, Commander T. Spatial distribution of the sibling species of Anopheles gambiae sensu lato (Diptera: Culicidae) and malaria prevalence in Bayelsa State, Nigeria. Parasites Vectors. 2014 Dec;7(1):32.

124. Figueiredo LTM. West Nile virus infection in Brazil. Rev Soc Bras Med Trop [Internet]. 2019 [cited 2025 Jul 22];52. Available from: http://www.scielo.br/scielo.php?script=sci_arttext&pid=S0037-86822019000100501&tlng=en

125. Odigie AE, Stufano A, Schino V, Zarea AAK, Ndiana LA, Mrenoshki D, et al. West Nile Virus Infection in Occupational Settings—A Systematic Review. Pathogens. 2024 Feb 9;13(2):157.

126. Abdullahi IN, Emeribe AU, Ghamba PE, Omosigho PO, Bello ZM, Oderinde BS, et al. Distribution pattern and prevalence of West Nile virus infection in Nigeria from 1950 to 2020: a systematic review. Epidemiol Health. 2020;42:e2020071.

127. Silva NIO, Sacchetto L, De Rezende IM, Trindade G de S, LaBeaud AD, De Thoisy B, et al. Recent sylvatic yellow fever virus transmission in Brazil: the news from an old disease. Virology journal. 2020;17:1–12.

128. Onoja AB, Omatola AC, Maiga M, Gadzama IS. Recurrent Episodes of Some Mosquito-Borne Viral Diseases in Nigeria: A Systematic Review and Meta-Analysis. Pathogens. 2022;11(10):1162.

129. Olaoye I, Fatiregun AA, Adejugbagbe AM, Dosumu M, Opeyemi E, Mahmud Z, et al. Characterization of Yellow Fever Suspected Cases and Confirmed Outbreaks in a Southwest State, Nigeria, 2021. Journal of Health Sciences & Surveillance System. 2022;10(4):420–8.

130. Nwachukwu William E, Oladejo J, Ofoegbunam CM, Anueyiagu C, Dogunro F, Etiki SO, et al. Epidemiological description of and response to a large yellow fever outbreak in Edo state Nigeria, September 2018-January 2019. BMC Public Health. 2022;22(1):1644.

131. Pielnaa P, Al-Saadawe M, Saro A, Dama MF, Zhou M, Huang Y, et al. Zika virus-spread, epidemiology, genome, transmission cycle, clinical manifestation, associated challenges, vaccine and antiviral drug development. Virology. 2020 Apr;543:34–42.

132. Basu R, Tumban E. Zika Virus on a Spreading Spree: what we now know that was unknown in the 1950’s. Virol J [Internet]. 2016 Dec [cited 2025 Jul 22];13(1). Available from: http://virologyj.biomedcentral.com/articles/10.1186/s12985-016-0623-2

133. Macnamara FN, Horn DW, Porterfield JS. Yellow fever and other arthropod-borne viruses; a consideration of two serological surveys made in South Western Nigeria. Trans R Soc Trop Med Hyg. 1959 Mar;53(2):202–12.

134. Fagbami AH. Zika virus infections in Nigeria: virological and seroepidemiological investigations in Oyo State. J Hyg (Lond). 1979 Oct;83(2):213–9.

135. Mathé P, Egah DZ, Müller JA, Shehu NY, Obishakin ET, Shwe DD, et al. Low Zika virus seroprevalence among pregnant women in North Central Nigeria, 2016. J Clin Virol. 2018 Aug;105:35–40.

136. Wales A, Mackintosh A. JMM Profile: Bacillus anthracis. Journal of Medical Microbiology. 2023;72(8):001747.

137. Carlson CJ, Kracalik IT, Ross N, Alexander KA, Hugh-Jones ME, Fegan M, et al. The global distribution of Bacillus anthracis and associated anthrax risk to humans, livestock and wildlife. Nature microbiology. 2019;4(8):1337–43.

138. Robinson TP, Thornton PK, Franceschini G, Kruska RL, Chiozza F, Notenbaert A, et al. Global livestock production systems [Internet]. Food and Agriculture Organization of the United Nations (FAO) and International Livestock Research Institute (ILRI),; 2011 [cited 2023 Aug 18]. Available from: https://www.fao.org/3/i2414e/i2414e.pdf

139. Blackburn JK, Odugbo MO, Van Ert M, O’Shea B, Mullins J, Perreten V, et al. Bacillus anthracis Diversity and Geographic Potential across Nigeria, Cameroon and Chad: Further Support of a Novel West African Lineage. PLoS Negl Trop Dis. 2015;9(8):e0003931.

140. Pitassi LHU, De Paiva Diniz PPV, Scorpio DG, Drummond MR, Lania BG, Barjas-Castro ML, et al. Bartonella spp. Bacteremia in Blood Donors from Campinas, Brazil. Walker DH, editor. PLoS Negl Trop Dis. 2015 Jan 15;9(1):e0003467.

141. Saengsawang P, Kaewmongkol G, Phoosangwalthong P, Chimnoi W, Inpankaew T. Detection of zoonotic Bartonella species in ticks and fleas parasitizing free-ranging cats and dogs residing in temples of Bangkok, Thailand. Veterinary Parasitology: Regional Studies and Reports. 2021 Jul;25:100612.

142. Kamani J, Baneth G, Mitchell M, Mumcuoglu KY, Gutiérrez R, Harrus S. Bartonella species in bats (Chiroptera) and bat flies (Nycteribiidae) from Nigeria, West Africa. Vector Borne Zoonotic Dis. 2014 Sep;14(9):625–32.

143. Warfel JM, Beren J, Kelly VK, Lee G, Merkel TJ. Nonhuman primate model of pertussis. Infection and immunity. 2012;80(4):1530–6.

144. Sun GQ, Li MT, Zhang J, Zhang W, Pei X, Jin Z. Transmission dynamics of brucellosis: Mathematical modelling and applications in China. Computational and Structural Biotechnology Journal. 2020;18:3843–60.

145. Tuon FF, Gondolfo RB, Cerchiari N. Human-to-human transmission of Brucella - a systematic review. Trop Med Int Health. 2017 May;22(5):539–46.

146. Li N, Yu F, Peng F, Zhang X, Jia B. Probable sexual transmission of brucellosis. IDCases. 2020;21:e00871.

147. Alavi SM, Motlagh ME. A Review of Epidemiology, Diagnosis and Management of Brucellosis for General Physicians Working in the Iranian Health Network. Jundishapur J Microbiol. 2012 Apr 1;5(2):384–7.

148. Akinyemi KO, Fakorede CO, Amisu KO, Wareth G. Human and Animal Brucellosis in Nigeria: A Systemic Review and Meta-Analysis in the Last Twenty-One Years (2001-2021). Vet Sci. 2022 Jul 26;9(8):384.

149. Dvorak GD, Spickler AR. Glanders. javma. 2008 Aug 15;233(4):570–7.

150. Torres AG. Glanders: An ancient and emergent disease with no vaccine or treatment on site. PLoS Negl Trop Dis. 2025 Jun;19(6):e0013160.

151. Akinniyi OO, Lawal TR, Rufai N, Jolayemi KO, Amaje J. Horse handlers’ knowledge, attitudes, and perceptions of African horse sickness in South-West, Nigeria. Sci Rep. 2025 Jul 1;15(1):21880.

152. Okorie-Kanu OJ, Anyanwu MU, Nwobi OC, Tambe-Ebot RY, Ikenna-Ezeh NH, Okolo CC, et al. Prevalence, Antimicrobial Resistance, and Virulence Potential of Staphylococcus aureus in Donkeys from Nigeria. Antibiotics (Basel). 2025 Apr 29;14(5):453.

153. Wiersinga WJ, Virk HS, Torres AG, Currie BJ, Peacock SJ, Dance DAB, et al. Melioidosis. Nat Rev Dis Primers [Internet]. 2018 Feb 1 [cited 2025 Jul 22];4(1). Available from: https://www.nature.com/articles/nrdp2017107

154. Orababa OQ, Adesida SA, Peters RF, AbdulGanniyu Z, Olakojo O, Abioye A. Showing the limitations of available phenotypic assays to detect Burkholderia pseudomallei from clinical specimens in Nigeria. Access Microbiology [Internet]. 2023 Oct 1 [cited 2025 Jul 29];5(10). Available from: https://www.microbiologyresearch.org/content/journal/acmi/10.1099/acmi.0.000604.v5

155. Veronese P, Dodi I. Campylobacter jejuni/coli Infection: Is It Still a Concern? Microorganisms. 2024 Dec 23;12(12):2669.

156. Audu BJ, Norval S, Bruno L, Meenakshi R, Marion M, Forbes KJ. Genomic diversity and antimicrobial resistance of Campylobacter spp. from humans and livestock in Nigeria. J Biomed Sci. 2022 Jan 24;29(1):7.

157. Begum S, Sekar M, Gunaseelan L, Gawande M, Suganya G, Malar PAS, et al. Molecular identification of Campylobacter jejuni and coli from chicken, calves and dogs to determine its potential threat on human being. Vet World. 2015 Dec;8(12):1420–3.

158. Beeckman DSA, Vanrompay DCG. Zoonotic Chlamydophila psittaci infections from a clinical perspective. Clinical Microbiology and Infection. 2009 Jan;15(1):11–7.

159. Wallensten A, Fredlund H, Runehagen A. Multiple human-to-human transmission from a severe case of psittacosis, Sweden, January–February 2013. Eurosurveillance [Internet]. 2014 Oct 23 [cited 2025 Jul 22];19(42). Available from: https://www.eurosurveillance.org/content/10.2807/1560-7917.ES2014.19.42.20937

160. Wang J, Wang B, Xiao J, Chen Y, Wang C. *Chlamydia psittaci* : A zoonotic pathogen causing avian chlamydiosis and psittacosis. Virulence. 2024 Dec 31;15(1):2428411.

161. Sheng Y, Jin L ying, Li N, Zhang Y, Shi Y jun. Global prevalence of psittacosis in outbreaks: a systematic review and meta-analysis. BMC Public Health. 2025 May 31;25(1):2010.

162. Dembek ZF, Mothershead JL, Owens AN, Chekol T, Wu A. Psittacosis: An Underappreciated and Often Undiagnosed Disease. Pathogens. 2023 Sep 15;12(9):1165.

163. eBird. Bird List (Nigeria) [Internet]. 2025 [cited 2025 Jul 30]. Available from: https://ebird.org/region/NG/bird-list

164. Koehler LM, Kloppert B, Hamann HP, El-Sayed A, Zschöck M. Comprehensive literature review of the sources of infection and transmission routes of Coxiella burnetii, with particular regard to the criteria of “evidence-based medicine.” Comparative Immunology, Microbiology and Infectious Diseases. 2019 Jun;64:67–72.

165. Körner S, Makert GR, Ulbert S, Pfeffer M, Mertens-Scholz K. The Prevalence of Coxiella burnetii in Hard Ticks in Europe and Their Role in Q Fever Transmission Revisited—A Systematic Review. Front Vet Sci [Internet]. 2021 Apr 26 [cited 2025 Jul 15];8. Available from: https://www.frontiersin.org/articles/10.3389/fvets.2021.655715/full

166. Celina SS, Cerný J. Coxiella burnetii in ticks, livestock, pets and wildlife: A mini-review. Front Vet Sci [Internet]. 2022 Nov 11 [cited 2025 Jul 15];9. Available from: https://www.frontiersin.org/articles/10.3389/fvets.2022.1068129/full

167. Ghanem-Zoubi N, Paul M. Q fever during pregnancy: a narrative review. Clinical Microbiology and Infection. 2020 Jul;26(7):864–70.

168. Cadmus S, Salam SP, Adesokan HK, Akporube K, Ola-Daniel F, Awosanya EJ. Seroprevalence of brucellosis and Q fever infections amongst pastoralists and their cattle herds in Sokoto State, Nigeria. PLoS One. 2021;16(7):e0254530.

169. Czepiel J, Dróżdż M, Pituch H, Kuijper EJ, Perucki W, Mielimonka A, et al. Clostridium difficile infection. European Journal of Clinical Microbiology & Infectious Diseases. 2019;38:1211–21.

170. Leffler DA, Lamont JT. Clostridium difficile infection. New England Journal of Medicine. 2015;372(16):1539–48.

171. Muscat M, Gebrie B, Efstratiou A, Datta SS, Daniels D. Diphtheria in the WHO European Region, 2010 to 2019. Eurosurveillance. 2022;27(8):2100058.

172. Gori M, Bolzoni L, Scaltriti E, Andriani L, Marano V, Morabito F, et al. *Listeria monocytogenes* Transmission from Donated Blood to Platelet Transfusion Recipient, Italy. Emerg Infect Dis [Internet]. 2023 Oct [cited 2025 Jul 22];29(10). Available from: https://wwwnc.cdc.gov/eid/article/29/10/23-0746_article

173. Hennebique A, Boisset S, Maurin M. Tularemia as a waterborne disease: a review. Emerging microbes & infections. 2019;8(1):1027–42.

174. Ombugadu A, Jibril A, Mwansat G, Njila H, Attah A, Pam V, et al. Composition and Distribution of Mosquito Vectors in a Peri-Urban Community Surrounding an Institution of Learning in Lafia Metropolis, Nasarawa State, Central Nigeria. Journal of Zoological Research| Volume. 2022;4(03).

175. WHO. WHO guidelines on tularaemia: epidemic and pandemic alert and response. 2007;

176. Harr B, Karakoc E, Neme R, Teschke M, Pfeifle C, Pezer Ž, et al. Genomic resources for wild populations of the house mouse, Mus musculus and its close relative Mus spretus. Sci Data. 2016 Sep 13;3:160075.

177. The IUCN Red List of Threatened Species [Internet]. [cited 2023 Sep 22]. Available from: https://www.iucnredlist.org

178. Leptospirosis in Humans. In: Current Topics in Microbiology and Immunology [Internet]. Berlin, Heidelberg: Springer Berlin Heidelberg; 2015 [cited 2025 Jul 15]. p. 65–97. Available from: https://link.springer.com/10.1007/978-3-662-45059-8_5

179. Benacer D, Woh PY, Mohd Zain SN, Amran F, Thong KL. Pathogenic and Saprophytic *Leptospira* Species in Water and Soils from Selected Urban Sites in Peninsular Malaysia. Microb Environ. 2013;28(1):135–40.

180. Abiayi EA, Itelima JU, Onwuliri FC, Udechukwu CC, Jolayemi KO, Abiayi DC, et al. Pathogenic Leptospira interrogans in Jos North Abattoir, Nigeria: Occurrence, serology, and molecular characterization. Comparative Immunology, Microbiology and Infectious Diseases. 2024 Sep;112:102223.

181. Charlier C, Disson O, Lecuit M. Maternal-neonatal listeriosis. Virulence. 2020 Dec 31;11(1):391–7.

182. Smith JP, Cohen T, Dowdy D, Shrestha S, Gandhi NR, Hill AN. Quantifying Mycobacterium tuberculosis transmission dynamics across global settings: a systematic analysis. American journal of epidemiology. 2023;192(1):133–45.

183. Guarner J. Buruli ulcer: review of a neglected skin mycobacterial disease. Journal of clinical microbiology. 2018;56(4):10–1128.

184. Merritt RW, Walker ED, Small PL, Wallace JR, Johnson PD, Benbow ME, et al. Ecology and transmission of Buruli ulcer disease: a systematic review. PLoS neglected tropical diseases. 2010;4(12):e911.

185. Rabiu I. Department of Microbiology, School of Science and Information Technology, Skyline University Nigeria. Buruli Ulcer (Mycobacterium ulcerans infection) in Nigeria: an update on the disease burden in Nigeria, J Clinical and Medical Research and Studies. 2(1).

186. Stephens DS, Greenwood B, Brandtzaeg P. Epidemic meningitis, meningococcaemia, and Neisseria meningitidis. The Lancet. 2007;369(9580):2196–210.

187. Ladhani SN, Lucidarme J, Parikh SR, Campbell H, Borrow R, Ramsay ME. Meningococcal disease and sexual transmission: urogenital and anorectal infections and invasive disease due to Neisseria meningitidis. The Lancet. 2020;395(10240):1865–77.

188. Lee Y, Kim SI, Yi Y sun, Lee H, Hwang JH, Park EC, et al. Transmission Electron Microscopy Confirmation of *Orientia tsutsugamushi* in Human Bile. Emerg Infect Dis. 2020 Dec;26(12):3101–3.

189. Musa TH, Ahmad T, Wana MN, Li W, Musa HH, Sharun K, et al. The epidemiology, diagnosis and management of scrub typhus disease in China. Human Vaccines & Immunotherapeutics. 2021 Oct 3;17(10):3795–805.

190. Kim G, Ha NY, Min CK, Kim HI, Yen NTH, Lee KH, et al. Diversification of Orientia tsutsugamushi genotypes by intragenic recombination and their potential expansion in endemic areas. Small PLC, editor. PLoS Negl Trop Dis. 2017 Mar 1;11(3):e0005408.

191. Elliott I, Pearson I, Dahal P, Thomas NV, Roberts T, Newton PN. Scrub typhus ecology: a systematic review of Orientia in vectors and hosts. Parasites Vectors. 2019 Dec;12(1):513.

192. Ma T, Hao M, Chen S, Ding F. The current and future risk of spread of Leptotrombidium deliense and Leptotrombidium scutellare in mainland China. Science of The Total Environment. 2022 Oct;843:156986.

193. Farr TG, Rosen PA, Caro E, Crippen R, Duren R, Hensley S, et al. The shuttle radar topography mission. Reviews of geophysics. 2007;45(2).

194. Afema JA, Byarugaba DK, Shah DH, Atukwase E, Nambi M, Sischo WM. Potential Sources and Transmission of Salmonella and Antimicrobial Resistance in Kampala, Uganda. Butaye P, editor. PLoS ONE. 2016 Mar 21;11(3):e0152130.

195. Hoelzer K, Moreno Switt A, Wiedmann M. Animal contact as a source of human non-typhoidal salmonellosis. Vet Res. 2011;42(1):34.

196. Akinyemi KO, Oyefolu AOB, Mutiu WB, Iwalokun BA, Ayeni ES, Ajose SO, et al. Typhoid Fever: Tracking the Trend in Nigeria. Am J Trop Med Hyg. 2018 Sep;99(3_Suppl):41–7.

197. Nelder MP, Russell CB, Johnson S, Li Y, Cronin K, Warshawsky B, et al. Assessing human exposure to spotted fever and typhus group rickettsiae in Ontario, Canada (2013–2018): a retrospective, cross-sectional study. BMC Infect Dis [Internet]. 2020 Dec [cited 2025 Jul 22];20(1). Available from: https://bmcinfectdis.biomedcentral.com/articles/10.1186/s12879-020-05244-8

198. Adamu A, Reyer F, Lawal N, Hassan AJ, Imam MU, Bello MB, et al. Aetiologies of bacterial tick-borne febrile illnesses in humans in Africa: diagnostic limitations and the need for improvement. Front Med (Lausanne). 2024;11:1419575.

199. Kamani J, Baneth G, Mumcuoglu KY, Waziri NE, Eyal O, Guthmann Y, et al. Molecular detection and characterization of tick-borne pathogens in dogs and ticks from Nigeria. PLoS Negl Trop Dis. 2013;7(3):e2108.

200. Reye AL, Arinola OG, Hübschen JM, Muller CP. Pathogen prevalence in ticks collected from the vegetation and livestock in Nigeria. Appl Environ Microbiol. 2012 Apr;78(8):2562–8.

201. Farshad S, Sheikhi R, Japoni A, Basiri E, Alborzi A. Characterization of Shigella strains in Iran by plasmid profile analysis and PCR amplification of ipa genes. Journal of clinical microbiology. 2006;44(8):2879–83.

202. Yismaw O, Negeri C, Kassu A. A five-year antimicrobial resistance pattern observed in Shigella species isolated from stool samples in Gondar University Hospital, northwest Ethiopia. Ethiopian Journal of Health Development. 2006;20(3).

203. Zafar MA, Wang Y, Hamaguchi S, Weiser JN. Host-to-Host Transmission of Streptococcus pneumoniae Is Driven by Its Inflammatory Toxin, Pneumolysin. Cell Host & Microbe. 2017 Jan;21(1):73–83.

204. Deen J, Mengel MA, Clemens JD. Epidemiology of cholera. Vaccine. 2020;38:A31–40.

205. Richterman A, Sainvilien DR, Eberly L, Ivers LC. Individual and household risk factors for symptomatic cholera infection: a systematic review and meta-analysis. The Journal of infectious diseases. 2018;218(suppl_3):S154–64.

206. Sabina Y, Rahman A, Ray RC, Montet D. *Yersinia enterocolitica*: Mode of Transmission, Molecular Insights of Virulence, and Pathogenesis of Infection. Journal of Pathogens. 2011;2011:1–10.

207. Okwori AEJ, Martínez PO, Fredriksson-Ahomaa M, Agina SE, Korkeala H. Pathogenic Yersinia enterocolitica 2/O:9 and Yersinia pseudotuberculosis 1/O:1 strains isolated from human and non-human sources in the Plateau State of Nigeria. Food Microbiology. 2009 Dec;26(8):872–5.

208. Lombin LH, Adesiyun AA, Agbonlahor DE, Kwaga JK. Isolation of Yersinia species from pigs in Nigeria. Vet Rec. 1985 Oct 5;117(14):364.

209. Agbonlahor DE, Adesiyun AA, Kwaga JK, Lombin LH. Colonial, biochemical and serological characteristics of Yersinia species isolated from animals in Nigeria. Rev Elev Med Vet Pays Trop. 1985;38(4):416–22.

210. Eisen RJ, Bearden SW, Wilder AP, Montenieri JA, Antolin MF, Gage KL. Early-phase transmission of Yersinia pestis by unblocked fleas as a mechanism explaining rapidly spreading plague epizootics. Proceedings of the National Academy of Sciences. 2006;103(42):15380–5.

211. Hinnebusch BJ, Jarrett CO, Bland DM. “Fleaing” the plague: adaptations of Yersinia pestis to its insect vector that lead to transmission. Annual review of microbiology. 2017;71:215–32.

212. Omitola OO, Taylor-Robinson AW. Emerging and re-emerging bacterial zoonoses in Nigeria: current preventive measures and future approaches to intervention. Heliyon. 2020;6(6).

213. Ugbomoiko U, Obiamiwe B. Distribution and incidence of ectoparasites on small mammals in a rainforest belt of southern Nigeria. Angewandte parasitologie. 1991;32(3):143–8.

214. Banda A, Gandiwa E, Muposhi VK, Muboko N. Ecological interactions, local people awareness and practices on rodent-borne diseases in Africa: A review. Acta Tropica. 2022;106743.

215. Neerinckx SB, Peterson AT, Gulinck H, Deckers J, Leirs H. Geographic distribution and ecological niche of plague in sub-Saharan Africa. International Journal of Health Geographics. 2008;7(1):1–12.

216. Craig P, Macpherson C. Parasitic helminths and zoonoses in Africa. Springer Science & Business Media; 2012. 281 p.

217. Nwafor KA, Onyali I, John ON, Ikpeze I, Njoku IU, Oji ZA. Communicating Bionomics of Cyclops in Communal Waters and their Guinea Worm Infection Status in Ebonyi State, Nigeria Fifteen Years After it Was Declared Guinea Worm Free. International Journal of Entomology and Nematology Research. 2022;6(1):11–28.

218. Adewole S, Hassan A. The impact of guinea worm disease on school attendance in Akoko, Ondo State, Nigeria. African Scientist. 2021;4(3).

219. World Health Organization. Lymphatic filariasis: a handbook of practical entomology for national lymphatic filariasis elimination programmes. World Health Organization; 2013. Report No.: 9241505648.

220. Eneanya OA, Fronterre C, Anagbogu I, Okoronkwo C, Garske T, Cano J, et al. Mapping the baseline prevalence of lymphatic filariasis across Nigeria. Parasites & vectors. 2019;12(1):1–13.

221. Federal Ministry of Health Nigeria. Neglected Tropical Diseases Nigeria Multi-Year Master Plan 2015–2020. 2015;

222. Bhosale NK, Parija SC. Balamuthia mandrillaris: An opportunistic, free-living ameba - An updated review. Trop Parasitol. 2021;11(2):78–88.

223. Basavaraju SV, Kuehnert MJ, Zaki SR, Sejvar JJ. Encephalitis caused by pathogens transmitted through organ transplants, United States, 2002-2013. Emerg Infect Dis. 2014 Sep;20(9):1443–51.

224. Siddiqui R, Khan NA. Balamuthia amoebic encephalitis: an emerging disease with fatal consequences. Microb Pathog. 2008 Feb;44(2):89–97.

225. Schuster FL, Dunnebacke TH, Booton GC, Yagi S, Kohlmeier CK, Glaser C, et al. Environmental isolation of Balamuthia mandrillaris associated with a case of amebic encephalitis. J Clin Microbiol. 2003 Jul;41(7):3175–80.

226. Pérez-Pérez P, Reyes-Batlle M, Morchón R, Piñero JE, Lorenzo-Morales J. Isolation and Molecular Identification of Pathogenic Free-Living Amoebae from Environmental Samples in Tenerife, Canary Islands, Spain. ACS ES T Water. 2025 Jun 13;5(6):2861–9.

227. Gerace E, Presti VDML, Biondo C. Cryptosporidium infection: epidemiology, pathogenesis, and differential diagnosis. EuJMI. 2019 Dec;9(4):119–23.

228. Li J, Cui Z, Qi M, Zhang L. Advances in Cyclosporiasis Diagnosis and Therapeutic Intervention. Front Cell Infect Microbiol [Internet]. 2020 Feb 11 [cited 2025 Jul 25];10. Available from: https://www.frontiersin.org/article/10.3389/fcimb.2020.00043/full

229. Krumrie S, Capewell P, Smith-Palmer A, Mellor D, Weir W, Alexander CL. A scoping review of risk factors and transmission routes associated with human giardiasis outbreaks in high-income settings. Current Research in Parasitology & Vector-Borne Diseases. 2022;2:100084.

230. Beasley EA, Mahachi KG, Petersen CA. Possibility of Leishmania Transmission via Lutzomyia spp. Sand Flies Within the USA and Implications for Human and Canine Autochthonous Infection. Curr Trop Med Rep. 2022 Sep 21;9(4):160–8.

231. Reimann MM, Torres-Santos EC, Souza CSFD, Andrade-Neto VV, Jansen AM, Brazil RP, et al. Oral and Intragastric: New Routes of Infection by Leishmania braziliensis and Leishmania infantum? Pathogens. 2022 Jun 16;11(6):688.

232. Ngouateu OB, Dondji B. Leishmaniasis in Cameroon and neighboring countries: An overview of current status and control challenges. Curr Res Parasitol Vector Borne Dis. 2022;2:100077.

233. Han B, Pan G, Weiss LM. Microsporidiosis in Humans. Clin Microbiol Rev [Internet]. 2021 Dec 15 [cited 2025 Jul 22];34(4). Available from: https://journals.asm.org/doi/10.1128/CMR.00010-20

234. Graumans W, Jacobs E, Bousema T, Sinnis P. When Is a Plasmodium-Infected Mosquito an Infectious Mosquito? Trends Parasitol. 2020 Aug;36(8):705–16.

235. Ahmadpour E, Foroutan-Rad M, Majidiani H, Moghaddam SM, Hatam-Nahavandi K, Hosseini SA, et al. Transfusion-Transmitted Malaria: A Systematic Review and Meta-analysis. Open Forum Infectious Diseases [Internet]. 2019 Jul 1 [cited 2025 Jul 22];6(7). Available from: https://academic.oup.com/ofid/article/doi/10.1093/ofid/ofz283/5514070

236. Dawaki S, Al-Mekhlafi HM, Ithoi I, Ibrahim J, Atroosh WM, Abdulsalam AM, et al. Is Nigeria winning the battle against malaria? Prevalence, risk factors and KAP assessment among Hausa communities in Kano State. Malar J. 2016 Jul 8;15:351.

237. Ponte-Sucre A. An Overview of Trypanosoma brucei Infections: An Intense Host–Parasite Interaction. Front Microbiol [Internet]. 2016 Dec 26 [cited 2025 Jul 22];7. Available from: http://journal.frontiersin.org/article/10.3389/fmicb.2016.02126/full

238. Bucheton B, MacLEOD A, Jamonneau V. Human host determinants influencing the outcome of *Trypanosoma brucei gambiense* infections. Parasite Immunology. 2011 Aug;33(8):438–47.

239. De Kyvon MALC, Maakaroun-Vermesse Z, Lanotte P, Priotto G, Perez-Simarro P, Guennoc AM, et al. Congenital Trypanosomiasis in Child Born in France to African Mother. Emerg Infect Dis. 2016 May;22(5):935–7.

240. Zongo K, Emmanuel RT. Advancing diagnosis and treatment for human African trypanosomiasis in Nigeria: challenges and future directions. Front Trop Dis [Internet]. 2025 Jan 6 [cited 2025 Jul 21];5. Available from: https://www.frontiersin.org/articles/10.3389/fitd.2024.1503421/full

241. Alanazi A, Younas S, Ejaz H, Alruwaili M, Alruwaili Y, Mazhari BBZ, et al. Advancing the understanding of Naegleria fowleri: Global epidemiology, phylogenetic analysis, and strategies to combat a deadly pathogen. Journal of Infection and Public Health. 2025 Apr;18(4):102690.

242. Sanchez SG, Besteiro S. The pathogenicity and virulence of *Toxoplasma gondii*. Virulence. 2021 Dec 31;12(1):3095–114.

243. Karshima SN, Karshima MN. Human Toxoplasma gondii infection in Nigeria: a systematic review and meta-analysis of data published between 1960 and 2019. BMC Public Health. 2020 Jun 6;20(1):877.
